# A standardized framework to test event-based experiments

**DOI:** 10.3758/s13428-024-02508-y

**Published:** 2024-09-16

**Authors:** Alex Lepauvre, Rony Hirschhorn, Katarina Bendtz, Liad Mudrik, Lucia Melloni

**Affiliations:** 1 Neural Circuits, Consciousness and Cognition Research Group, Max Planck Institute of Empirical Aesthetics, Frankfurt am Main, Germany; 2 Donders Institute for Brain, Cognition and Behaviour, Radboud University Nijmegen, Nijmegen, 6500 HB the Netherlands; 3 Sagol School of Neuroscience, Tel-Aviv University, Tel Aviv, Israel; 4 Boston Children’s Hospital, Harvard Medical School, Boston, USA; 5 School of Psychological Sciences, Tel-Aviv University, Tel Aviv, Israel; 6 Department of Neurology, NYU Grossman School of Medicine, New York, USA; 7 Canadian Institute for Advanced Research (CIFAR), Brain, Mind, and Consciousness Program, Toronto, ON Canada

**Keywords:** Replication, Experimental methods, Pre-acquisition tests

## Abstract

The replication crisis in experimental psychology and neuroscience has received much attention recently. This has led to wide acceptance of measures to improve scientific practices, such as preregistration and registered reports. Less effort has been devoted to performing and reporting the results of systematic tests of the functioning of the experimental setup itself. Yet, inaccuracies in the performance of the experimental setup may affect the results of a study, lead to replication failures, and importantly, impede the ability to integrate results across studies. Prompted by challenges we experienced when deploying studies across six laboratories collecting electroencephalography (EEG)/magnetoencephalography (MEG), functional magnetic resonance imaging (fMRI), and intracranial EEG (iEEG), here we describe a framework for both testing and reporting the performance of the experimental setup. In addition, 100 researchers were surveyed to provide a snapshot of current common practices and community standards concerning testing in published experiments’ setups. Most researchers reported testing their experimental setups. Almost none, however, published the tests performed or their results. Tests were diverse, targeting different aspects of the setup. Through simulations, we clearly demonstrate how even slight inaccuracies can impact the final results. We end with a standardized, open-source, step-by-step protocol for testing (visual) event-related experiments, shared via protocols.io. The protocol aims to provide researchers with a benchmark for future replications and insights into the research quality to help improve the reproducibility of results, accelerate multicenter studies, increase robustness, and enable integration across studies.

**Supplementary Information:**

The online version contains supplementary material available at 10.3758/s13428-024-02508-y.

## Introduction

The remarkable progress of experimental human neuroscience in recent decades, fueled by the development of technologies to survey the brain non-invasively, has been partly overshadowed by the many examples of replication failures (e.g., Hirschhorn & Schonberg, 2024). Replication failures may stem from a number of factors (Open Science Collaboration, 2015), for instance, low standards of power calculations (Button et al., 2013; Ioannidis, 2005), the use of questionable statistical methods (e.g., Cumming, 2014; Wicherts et al., 2016), publication biases in favor of positive findings (Fanelli, 2012), or scarce description of the methods (Poldrack et al., 2008; Simmons et al., 2011).

The community has responded to those challenges by promoting better scientific practices that address those issues (Munafò et al., 2017). By now, determining sample sizes based on power analysis has become a common practice (e.g., Mumford & Nichols, 2008), journals also more routinely publish negative or null results (e.g., Baxter & Burwell, 2017), and preregistering the planned design and analyses is on the rise (e.g., AsPredicted, Open Science Framework). These procedures have had a large impact on the scientific community (Logg & Dorison, 2021; Protzko et al., 2023). One aspect that has received less attention in the empirical sciences, however, is the functioning of the experiment itself: does the experiment run as expected? While at first glance the reader may assume that experiments always run as planned and that if errors occur, they do not significantly impact the results, here we show a large variance among researchers when it comes to testing the experimental framework. Furthermore, we demonstrate that errors in the functioning of the experiment (e.g., the timing of the stimuli) can impact the results and their interpretation. Despite its importance, no standardized procedure for testing and reporting the quality of the experimental setups currently exists.

Standardized procedure**s** are becoming more relevant given the increased number of multi-lab studies (e.g., Frank et al., 2017; Melloni et al., 2021; Pavlov et al., 2021) and the increased availability of openly shared data. The large diversity of software and hardware poses a major challenge when integrating data across different laboratories, which often acquire data using different setup specifications. A similar issue arises when reusing openly shared data collected in various neuroscientific paradigms (Sejnowski et al., 2014). Without metadata describing the functioning of the experimental setup itself (variability in the presentation duration of the stimuli, reliability of the timestamps, etc.), integrating multiple datasets can pose problems, as it necessitates determining a priori how comparable the data are (Carp, 2012). For that, information about if and how the experiment was tested is important.

To demonstrate the need for a standardized testing framework, we first surveyed current practices in the field when it comes to testing and reporting the functioning of experimental setups in neuroscience. Testing practices varied among researchers, and many acknowledged discovering malfunctioning upon data collection. We then used simulations to demonstrate that even minor inaccuracies in hardware and software can alter results. Finally, we propose a standardized framework for testing and reporting the functioning of experimental setups for event-based designs. We provide an easy-to-use protocol, openly available in protocols.io.

### Common testing practices: A survey

To investigate current practices of testing experimental setups in behavioral and neural science, 100 psychologists and neuroscientists reported on studies they had recently carried out. The majority of respondents studied human participants (94/100), collected neural data (67/100), and were early-career researchers (40/100 graduate students, 36/100 postdoctoral/senior researchers, 16/100 principal investigators).

**Fig. 1:**
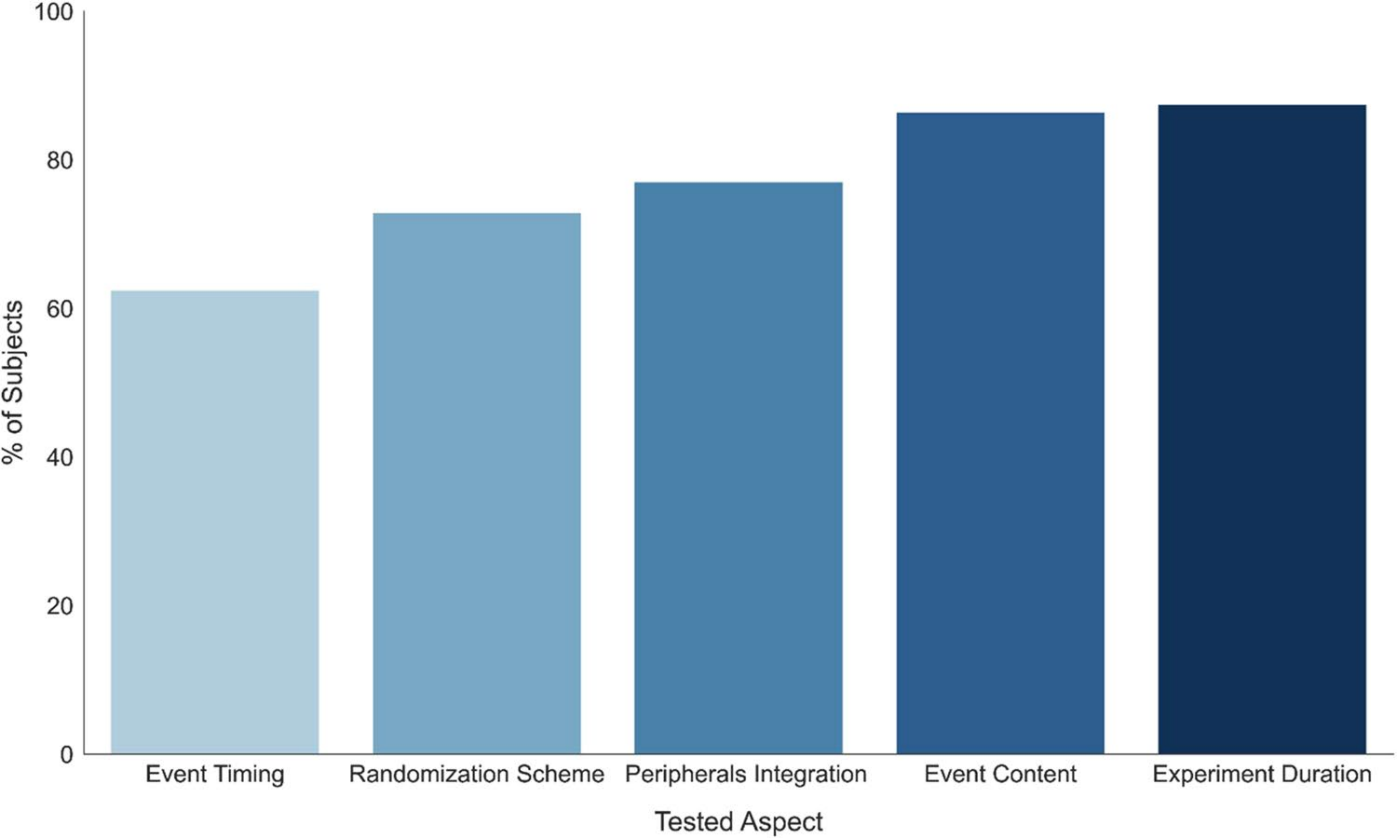
The type and frequency of pre-experimental tests, declared by researchers to have been conducted prior to their last published experiment ( N  = 96). Y -axis: percentage of respondents. X -axis: aspect of the experimental environment tested (selected from a list of options; see Supplementary Material 1 ). The terms "event timing" and "event content" are defined in Box 1

Almost all respondents reported testing the experimental setup prior to data acquisition in a large majority of experiments (91/100), while a few (5/100) tested only some experiments or never tested the setup before data collection (4/100). The aspects of the experimental setup tested varied greatly among researchers (Fig. 1). Most tested the overall duration of the experiment (84/96), while a smaller proportion tested the accuracy of the event timings (60/96; see Box 1). There was also considerable diversity in the methods used to test the experimental setup, both between researchers (e.g., manual checks [48/96], scripted checks ([1/96], both types ([47/96]) and within the same lab between experiments (when asked whether the tests were based on a protocol, 53/96 responded that each experiment was tested differently; see Supplementary Material 1 for survey and the full set of results).

Strikingly, a large proportion of researchers (64/100) reported noticing an issue after data collection that could have been avoided through prior testing. This reinforces the need for a streamlined procedure to benchmark the experimental setup (or *experimental environment*, as defined in Box 1), which could prevent the collection of unusable data and facilitate replication.

**Box 1** Definitions of terms

**Table:** 

**Fig. 2:**
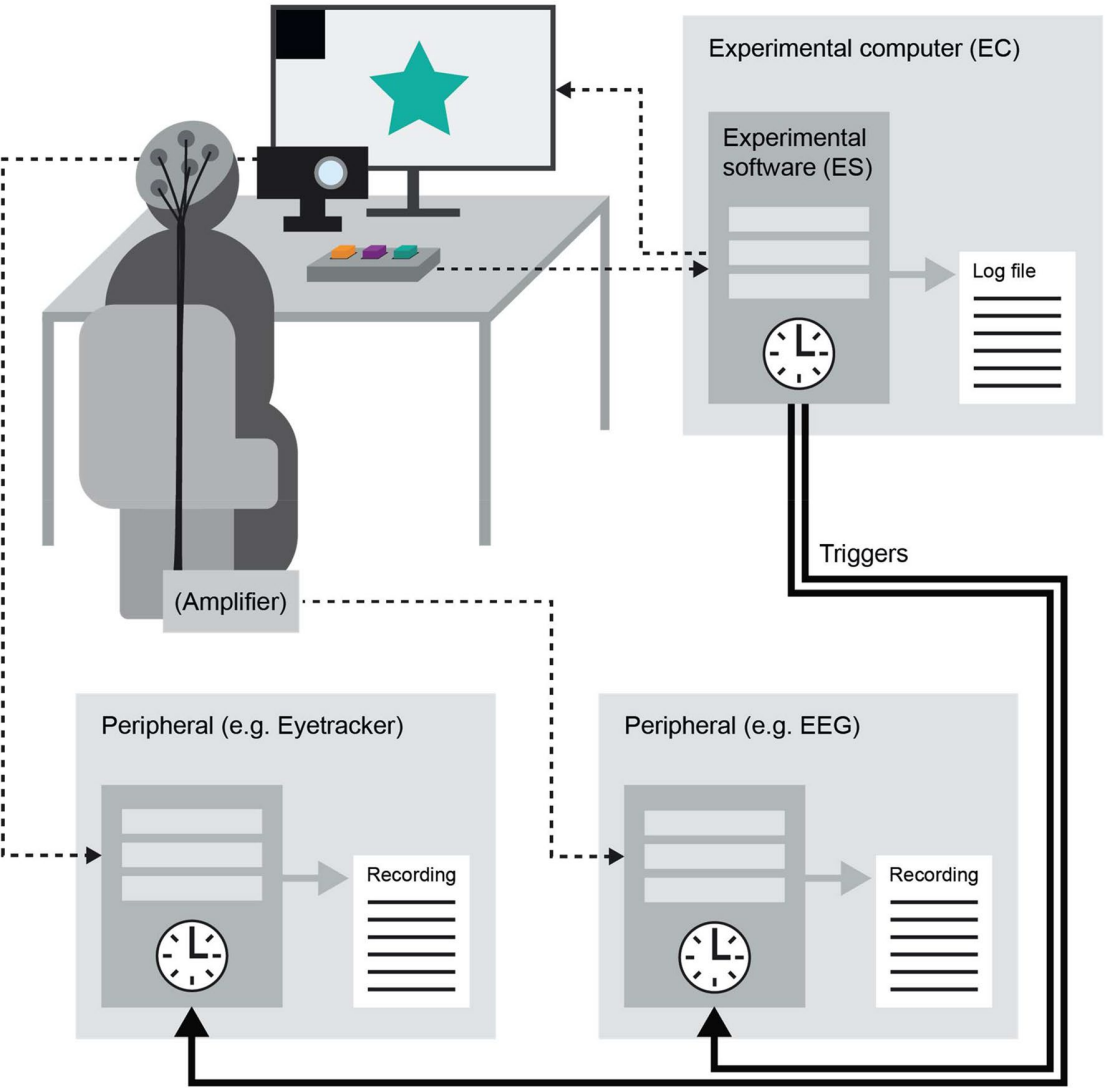
The image illustrates the connections between the various components and devices used in an example experiment. **Top left**: A participant sits in an experimental environment (see Box 1). They face a computer screen displaying a green star. Underneath is an eye-tracking device (black) and a response box. An EEG cap and amplifier are displayed as an example of neural measurement. **Top right**: The screen connects to the experimental computer which runs the experimental software (see Box 1). **Bottom** **right**: An example peripheral device recording neural data, connected to both the experimental computer (top right) and the EEG amplifier (top left). **Bottom** **left**: An example peripheral device recording eye-tracking data connected to both the eye-tracking camera (top left) and the experimental computer (top right). Clocks in each box indicate the device’s internal clock. *Dashed arrows* depict connections between components of independent devices. *Solid black arrows* represent connections sending triggers from the experimental computer to peripherals used for synchronization between recording devices.

**Box 1: Definitions**
*Event-based design:* an experimental design in which the participant is presented with specific stimuli (e.g., images, sounds) at prespecified times, to measure the reaction to those stimuli (e.g., behavior, neural activity, physiological response).
*Event:* refers to the presentation of a certain stimulus at a particular time in an event-based design.
*Event timing:* refers to the time during the experiment when an event of interest takes place in an event-based design.
*Event content:* refers to all aspects specifying an event, regardless of its timing during the experiment. For example, for a visual stimulus, its content comprises its identity (e.g., “a face”), location (e.g., “screen center”), and other features that differentiate it from the rest of the stimuli in the experiment and/or are relevant to the experimental conditions (e.g., orientation, color, luminance, size, presentation duration, belonging to a specific category of stimuli, task relevance, congruency with other events).
*Experimental design:* **r**efers to the desired scheme that dictates how many stimuli shall be presented from each category, the expected order in which they shall be presented, and their duration and timing. It specifies both the timing of the expected sequence of events and the event content.
*Experimental environment:* all hardware and software that is part of the experiment. This includes, but is not limited to, the software used to present stimuli and collect responses from the participant (*experimental software*), the computer on which the experimental software runs (*experimental computer*), and any device that the experimental software and the computer communicate to during the experiment (*peripherals*). For example, peripherals could include the screen on which a visual stimulus is presented, cameras that record the participant, and devices measuring neural and/or other physiological activity.
*Experimental computer (EC):* the computer on which the *experimental software* runs to present the participants with the *events* of the experiment.
*Experimental software (ES):* the software (e.g., Psychtoolbox, PsychoPy, Presentation) executing the experimental program on the *EC*.
*Log file:* all the information written to the *EC*’s disc through the *ES* during a single experimental run. This includes information about all events presented during the experiment (*event content* and *timing*) and all the measurements the *EC* recorded directly (e.g., mouse click, keyboard response) and indirectly (e.g., information reaching from *peripherals*). When the peripheral runs on its own internal clock (see Fig. 2), its output is recorded on a separate file. Thus, one experiment could have several output files.
*Peripherals:* all the hardware (and the software used to operate it) that are connected to the EC, or communicate with the ES in some way. We refer to two peripheral types: (1) peripheral devices with their own internal clock (e.g., neural imaging hardware and software), which communicate with the EC via triggers, and (2) devices that are run on the EC’s internal clock (e.g., response box, keyboard, computer mouse). All peripherals are part of the *experimental environment*.
*Experimental output:* all output files produced by devices that are included in the *experimental environment*. This includes all the output files of both the ES and peripherals.
*Triggers:* messages sent to/from the EC from/to peripherals, used in order to synchronize the peripherals and ECs.
*Controlled events:* any experimental *events* controlled by parameters predefined by the researcher, e.g., stimuli presented to the participant.
*Uncontrolled events:* any experimental *events* that depend on and are controlled by the participant (and not the researchers), e.g., participants’ motor responses.
*Events’ physical realization:* the actual occurrence of an event within the *experimental environment* (as opposed to the planning or logging of that event). The exact timing of the physical realization of an event can be determined by measuring the changes in the physical properties of the experiment setup (e.g., changes in luminance, or changes in decibels).
*Delay:* refers to constant temporal shift between the physical realization of an event and its recorded timestamp on an experimental device clock. For example, the timestamping of stimulus onsets recorded by EEG triggers is systematically delayed by 32 ms relative to the actual stimulus onsets (which can be inferred from the photodiode signal). Such delays have been discussed extensively in the M/EEG literature (Farzan et al., 2017; Pernet et al., 2018). By measuring these constant delays, they can easily be compensated for by shifting events' timestamps by the measured delay before data analysis (dedicated functions have been developed to do so, mne: epochs.shift_time, fieldtrip).
*Jitter*: refers to the varying temporal shift between the physical realization of an event and its recorded timestamp on an experimental device clock. Unlike the delay, jitters are not constant across trials and therefore cannot be easily compensated for.

**Fig. 3:**
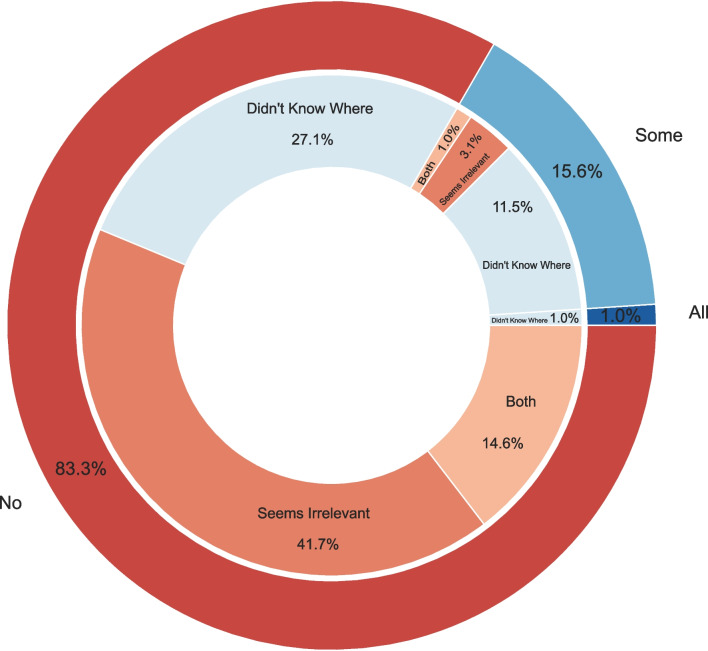
Reporting practices of researchers who declared testing their last published study ( N  = 96, since four respondents declared not testing their experiments at all). Outer circle: responses to “Did you report about the checks you performed and their results?” Red: respondents declaring not reporting the results of the tests ( N  = 80). Light blue: respondents declaring reporting the results of some tests ( N  = 15). Blue: one respondent who reported the results of all the performed tests. Inner circle: responses to “If you did not report the checks, why not?” Orange: because it was irrelevant ( N  = 3 out of the “some” category, N  = 40 out of the “no” category). Grayish-blue: because it wasn’t known where to report the results ( N  = 11 [“some” category], N  = 26 [“no” category], and 1 reporting the test). Tan: because it was irrelevant and not known where to report the results ( N  = 1 [“some” category], N  = 14 [“no” category])

Despite performing tests, results in publications (80/96) either because they considered reporting the results irrelevant (43/96) or because they did not know where to present them (38/96), or both (15/96; Figs. 3). Reporting practices, from reporting in the methods section, in the preregistration, in their lab book, or supplementary materials. A small proportion of researchers who never reported results (10/80) assumed that all published work had been thoroughly checked. However, as we have seen, the assumption that experimental tests and methods are consistent and error-free is not warranted, given the variations in testing procedures and the prevalence of errors identified retrospectively, during or after data collection. Thus, reporting of test results is crucial. If widely adopted, this practice would encourage more thorough testing, reduce errors, and enhance data accuracy across experiments and datasets.

### Simulations of experimental environment malfunction

The survey demonstrated that research practices for testing the experimental setup vary widely, and that if tests are conducted, they are often not reported. Yet, researchers acknowledged discovering inaccuracies after data acquisition which could have been prevented. How severe are those inaccuracies to warrant extra testing and reporting? We simulated inaccuracies in event content and event timing to demonstrate whether and how they can affect experimental results (Supplementary Material 2 for full simulation). For this demonstration, we focused on reaction time data and P1 event-related potential (ERP), as both are widely used measurements and highly susceptible to timing inaccuracies. Both behavioral and neural simulation results are generalizable to other ERP components and *uncontrolled events* (events evoked by participants’ responses; see Box 1). We simulated two experimental conditions, representing two stimulus groups (see Supplementary Material 2 for the complete procedure). The simulation followed a common experimental hypothesis of a difference between two conditions, in both the P1 average amplitude and mean reaction times. The difference between the two conditions was referred to as $\theta$. To simulate inaccuracies in event contents, we shuffled stimulus labels on some trials (between 2% and 40%); for inaccuracies in event timings, we introduced a jitter of a predefined duration (from 2 to 40 ms) on some trials (between 2% and 40%). Simulations show that content and timing inaccuracies considerably diminish and sometimes even obliterate statistical differences between experimental conditions.

**Fig. 4:**
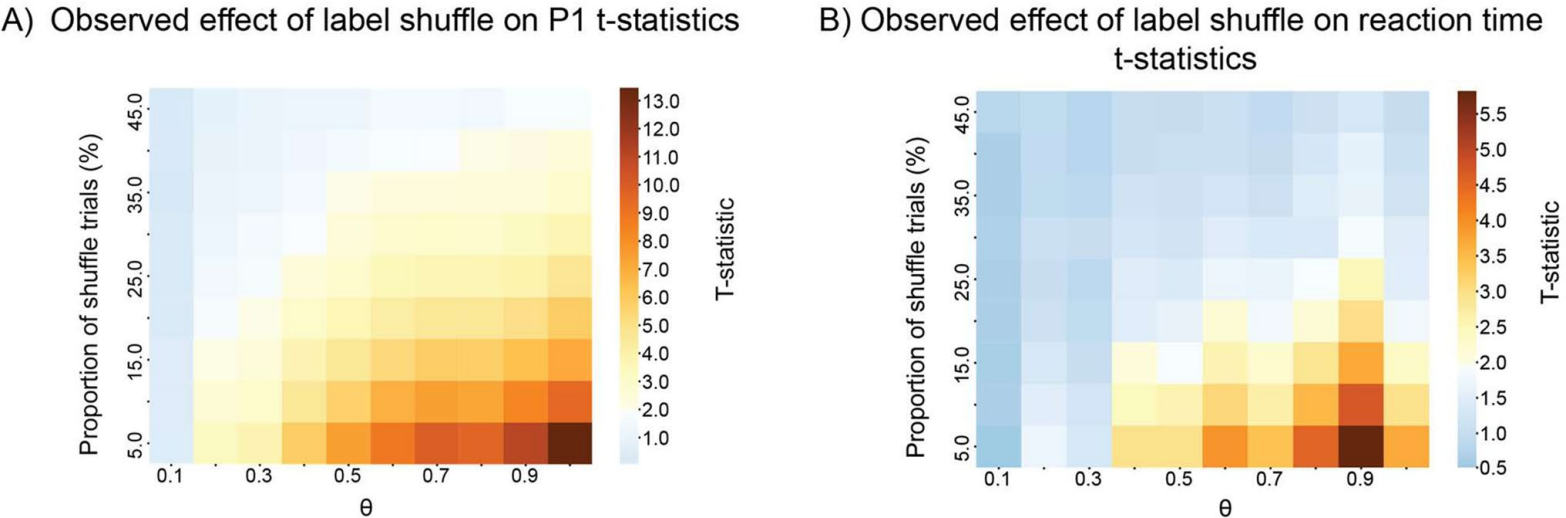
Effect of label shuffle on the P1 (A) and reaction time (B) *t*-statistic. The heatmap represents the observed *t*-statistic as a function of the simulated effect size (*x*-axis) and proportion of trials for which the labels were shuffled (*y*-axis). The color bar is centered on 1.96. Values below significance are colored in shades of blue, while values above significance are colored in shades of orange.

**Fig. 5:**
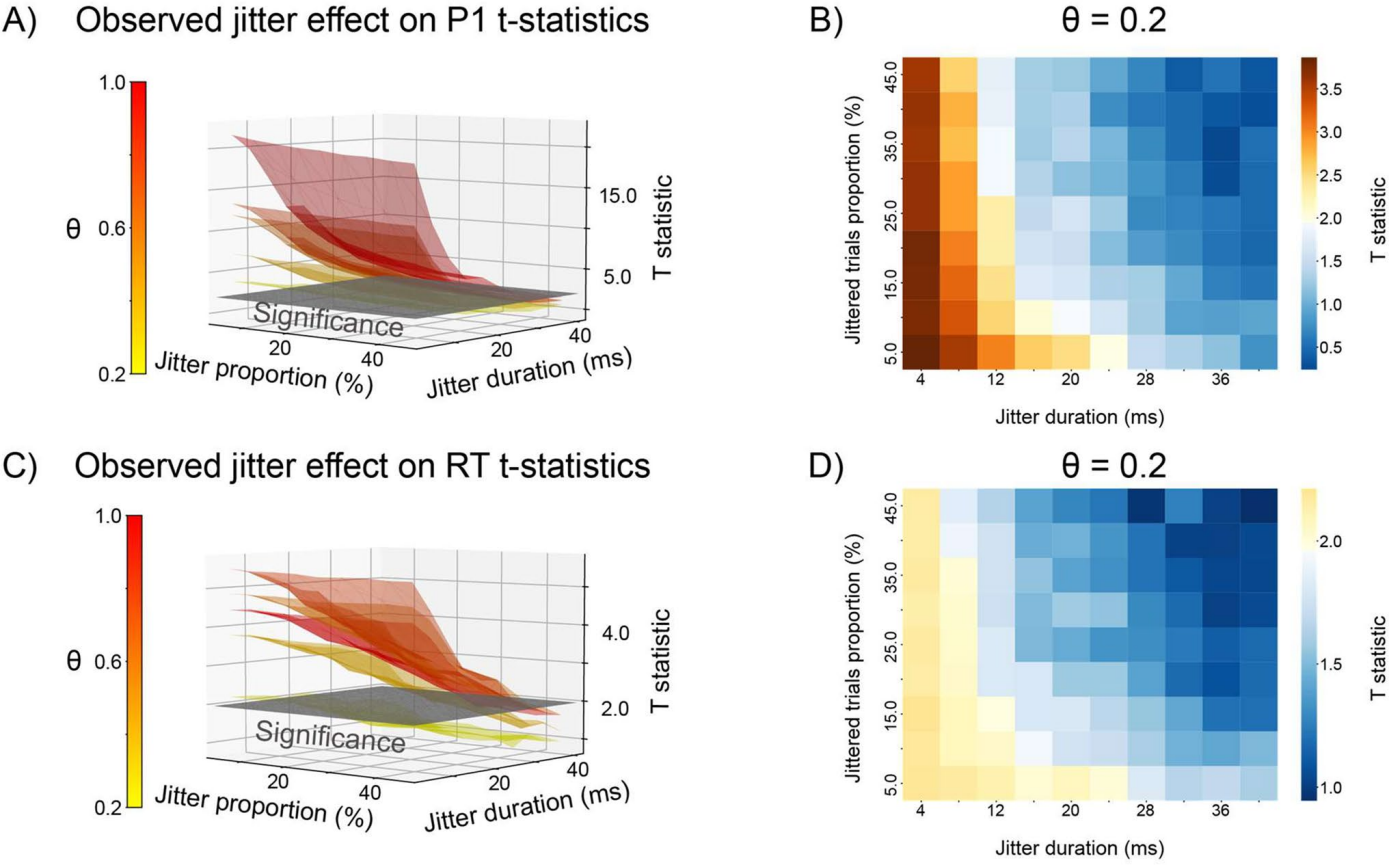
Effects of timing inaccuracies on *t*-statistic for ERPs (A, B) and reaction time (C, D). A, C 3D plot relating the proportion of jittered trials (*x*-axis), jitter duration (*y*-axis), and *t*-statistic (*z*-axis) as a function of effect size ($\uptheta$, color bar) for P1 amplitude and reaction times, respectively. Gray hyperplane depicts significance threshold of *t* = 1.96. An example at $\uptheta$ = 0.2 for P1 amplitude (**B**) and reaction time (**D**). The color bar centered on 1.96, values below significance are colored in shades of blue, while values above significance are colored in shades of orange

Figure 4A shows that at small effect sizes ($\theta \hspace{0.17em}$= 0.2), a small proportion of shuffled-label trials (20%) was enough to abolish a statistical effect in P1 amplitude. For the reaction time, the same was observed when 5% or more labels were shuffled across trials (Fig. 4B). Jitter in stimulus timing also significantly affected the measured statistic: Figure 5B shows that a jitter of 16 ms (a frame on a 60 Hz monitor) on 15% of the trials was enough to render P1 effects insignificant. For a 32 ms jitter, the same outcome was observed when 5% of the trials were affected. The effect of jitter was comparable for the reaction time: at $\theta$= 0.2, a 16 ms jitter affecting 15% of trials was sufficient to abolish the effect, and the same was observed at larger effect sizes ($\theta$= 0.5) with a 32 ms jitter (two frames) in 5% of trials (Fig. 5D).

Taken together, the simulation results reinforce the importance of testing the recorded stimulus contents, showcasing the effect that timing inaccuracies and imprecision of the hardware (experimental computer [*EC*]; the computer on which the experiment runs; Box 1) can have. Recent studies suggest that inaccuracies in modern experimental software (*ES*; e.g., PsychoPy: Brainard, 1997; Psychtoolbox: Peirce, 2007) are minimal (on the sub-millisecond level, Bridges et al., 2020). This is only the case when the experiment is run in an *ideal* experimental environment. Accordingly, proper testing is required to ensure that this is indeed the case. This is even more important given the variations in the interaction between the EC and the ES (for example, in Psychtoolbox, a screen flip can be missed if too many textures are open; see definition in Box 1). Thus, our simulation results highlight the need for standardized testing of the experimental environment. Here, we argue that performing a few basic tests can increase event-based experiment reproducibility, improve data integration across datasets, and ultimately minimize errors, increasing efficiency. Next, we describe a standardized framework of tests.

### Framework

We describe a standardized framework to benchmark the experimental environment in event-based designs including a standardized reporting protocol (protocols.io). The framework is aimed at helping researchers without imposing additional burdens on their standard experimental procedures. As such, it strikes a balance between exhaustiveness and ease of use. As a starting point, the framework is best suited for studies involving visual stimuli while collecting responses from participants (neural responses as well as behavior and eye-tracking data). Extension to other modalities (e.g., auditory, tactile) and response devices (e.g., microphone) will be needed. To illustrate the framework, we programmed a simple experiment in which we conducted all the tests, described step by step in a Jupyter notebook (see Supplemental Material 3). The notebook aims to help researchers in understanding the implementation of the framework, serving as an accessible resource that can be adapted for testing future experiments.

**Fig. 6:**
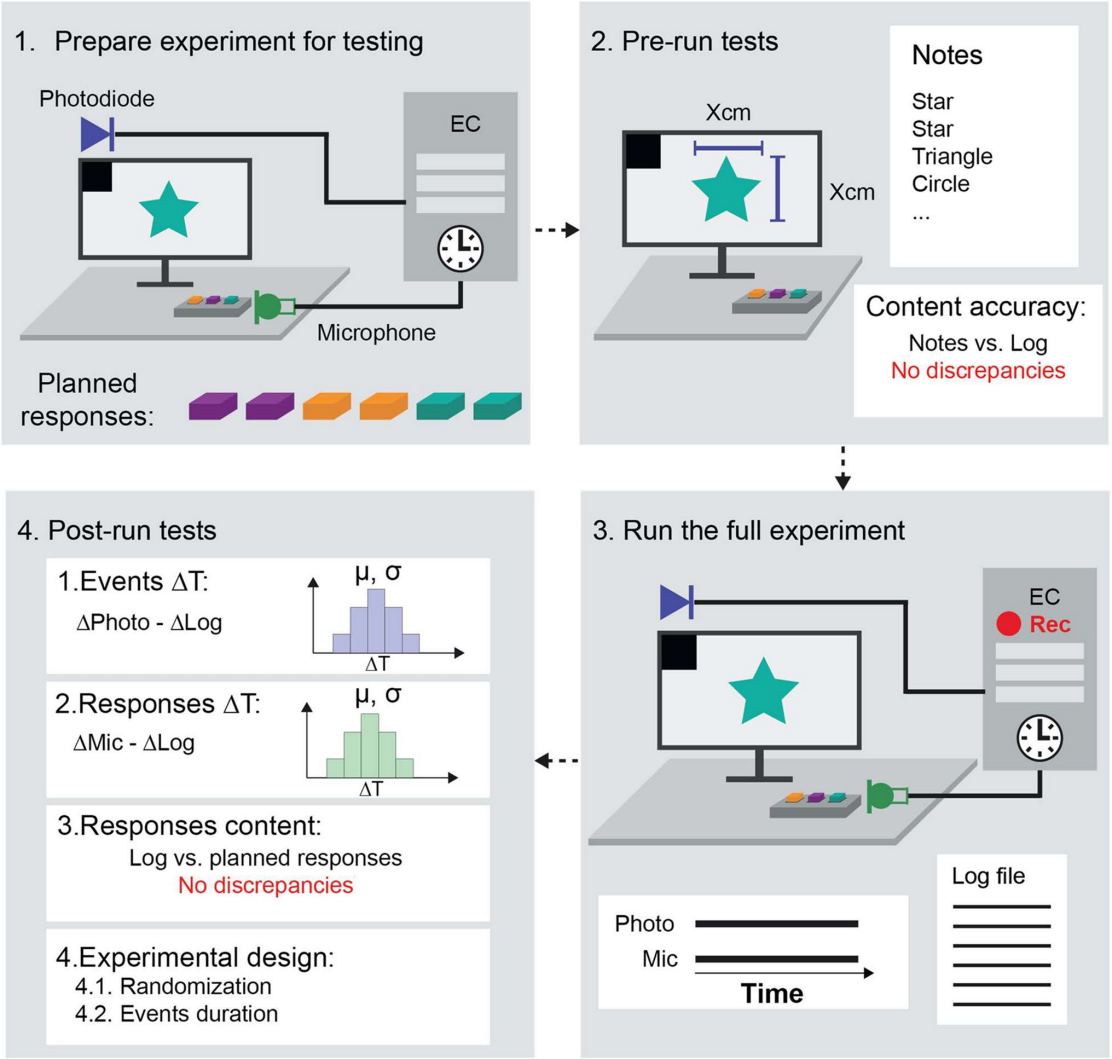
Flowchart of the implementation of the testing framework. (1) First, the experiment should be adjusted to present a photodiode square and record the sounds from a contact microphone measuring the sound produced by keyboard presses. In addition, a response sequence should be planned. (2) Then, a set of pre-run tests should be performed. The first consists of measuring the size of the stimuli in centimeters to compute the size of the observed stimuli in degrees of visual angles. In addition, several trials of the experiment should be run, manually annotating what was presented on the screen in each trial. These manual annotations should then be compared to the log file entries to ensure that the log file accurately records what was actually presented. (3) The experiment should then be run in full while recording the photodiode and microphone signals as well as the log file for offline analysis. (4) Then, the recorded photodiode signal should be compared to the log file to estimate event timing inaccuracies. The microphone signal should be compared to the log file response timestamping to assess the responses’ timestamping inaccuracies. In addition, the logged responses should be compared to the planned response sequence defined in step (1) to ensure that the log file accurately records the pressed buttons. Finally, the experimental design can be tested for correctness based on the log file and photodiode timing information

Each section starts with the motivation for testing a given aspect of the experimental environment, followed by the testing guidelines and the standardized reporting protocol (see protocols.io). A successful visual event-based experiment necessitates thorough testing and validation of four key aspects: (1) the completeness and accuracy of the log file regarding *event content*, (2) the same for *event timing*, (3) the alignment between actual events and the planned experimental design, and (4) the reliability of peripheral triggers. This ensures experimental integrity and comparability across different studies and laboratories. Testing typically involves running the entire experiment at least once in the final experimental setup, as the hardware significantly influences the precision and accuracy of the experiment. While these aspects are discussed separately, they can generally be tested in a single experiment run, unless specified otherwise. We provide a visual representation of the implementation of all steps of the framework (see Figs. 6).

## Validating the features of the controlled events

### Why test it?

In most visual experiments, standardizing the visual angle and eccentricity across participants is crucial. The initial step involves correctly setting up the visual angle and eccentricity in the experimental environment. This standardization is foundational for all subsequent tests and ensures that experimental events are presented under consistent conditions. This step is critical for both multi-lab studies and single-lab experiments, facilitating accurate replication by ensuring visual equivalence between original and replication setups.

#### How to test it?

First, three measurements should be obtained: the screen’s height and width in pixels (i.e., the screen resolution), the screen’s height and width in metric units, and the distance between the eye and the screen (in metric units). From the screen dimensions in centimeters and pixels, a conversion factor between pixels and centimeters should be computed as follows:

1 $$\begin{array}{ccc}c= \frac{{Height}_{cm}}{{Height}_{pixel}}& \text{or}& c= \frac{{Width}_{cm}}{{Width}_{pixel}}\end{array}$$

Using either the right or the left equation (i.e., the height or the width) should yield the same results, except for displays in which the pixel aspect ratio is not 1:1. In all other cases, inequality indicates measurement issues. After obtaining the conversion factor *c*, event sizes and offsets can be calculated in degrees of visual angles from screen pixels and size in metric units.

The next step is to measure the size and eccentricity of a specific experimental event. Once presented, the size and eccentricity of the visual event can be measured in metric units. Then, the inverse tangent can be used to calculate the vertical and horizontal visual angle of a stimulus using right-angle trigonometry:

2 $$\theta=2\times atan\left(\frac{\frac{size}2}{distance}\right)$$

where both size and distance are in metric units.

Similarly, the eccentricity should be calculated in visual angles by measuring the distance between the center of the screen and the center of the stimulus of interest (assuming fixation is in the middle of the screen) and applying the same formula (2).

Note that in the case that stimuli are off-centered, the measurement of the stimulus size in visual angles needs to be adjusted to account for the tilt between the screen and the eye. SR Research offers a free tool for visual angle calculation, for all types of stimuli (centered, one-sided, off-center; see www.sr-research.com/visual-angle-calculator/). When experimental events differ in size and eccentricity (e.g., stimuli displayed in different locations or differing in size), each such event should be measured at least once (per location, per size).

#### How to report it?

To report the relevant visual features in degrees of visual angle, the measured distance of the screen should be reported in centimeters (to describe the conditions under which the size of the stimuli was tested). For the stimuli, the measured height and width should be reported in degrees of visual angle. If different stimuli sizes are relevant to the experimental design, those should be measured and reported too. Similarly, if the stimuli are presented at a given eccentricity, both the expected and measured horizontal and vertical distance from the center of the screen should be reported (also in visual angles). Reporting both the horizontal and vertical visual angles from the expected center of the participant’s gaze provides the unique position of the stimuli. Thus, it is preferable to report the distance between the center of the stimulus and where participants are supposed to fixate (usually, the center of the screen).

## Testing the reliability of the log file event content

### Controlled events

#### Why test it?

For accurate analysis, researchers must ensure that the content of events presented to participants is correctly recorded in the log files (see Box 1). This involves verifying that logged events match the actual events presented. Errors in the experimental software (ES) or hardware (EC) can lead to incorrect logging, such as mislabeling stimulus categories or identities, which can significantly alter experimental results. Any discrepancies require rectifying and retesting until the log file accurately reflects the presented content. While systematically checking log files, especially for experiments with complex stimuli like videos, can be challenging and time-consuming, it is crucial for ensuring the validity, interpretability, and reproducibility of the results.

### How to test it?

Comparing the on-screen content with that of the log file where the content is documented requires running the experiment, ideally, from start to end (without participants), noting the event content presented on the screen (e.g., the stimulus identifier and relevant features such as orientation, location, color). Manually noting the content of each stimulus throughout the entire experiment might not be feasible, especially for experiments containing hundreds of trials. The compromise recommended here is to minimally check the content of each event condition at least once (though exhaustive testing is, of course, preferred). For example, suppose an experiment presents two stimulus groups (e.g., faces and objects) at four possible locations. In that case, the manual recording of event content during the experiment should at least cover a stimulus from each group appearing at each location once. By event condition, we refer to any feature relevant to the experimental design (e.g., category, location, task relevance, color, congruence). For designs with nested conditions (e.g., stimuli of different groups presented in different task-relevance conditions), a condition is understood as a combination of conditions (a task-relevant face constitutes a condition, and task-irrelevant faces another).

As events can be fast-paced, it might be impossible to mark them manually in real time. Therefore, we recommend following one of two options: One possibility is using external recording devices and software (cameras, microphones, or other recording software) to record the presented events, tagging them based on the recording’s playback. Importantly, the recording device needs to be external to the experimental environment (e.g., not a recording software running on the EC), as otherwise, it might interfere with the functioning of the experimental environment (as a screen-recording software is not expected to run in the data collection phase). Alternatively, the pace of the experiment could be slowed down for testing purposes in the ES such that the event content can be noted. The downside is that then two separate tests are required: one for testing the event content and a separate one for testing the reliability of the event timings (see next section).

The logged on-screen contents are then compared to the saved log files of the test run, expecting complete consistency between the two. Discrepancies point to malfunction of the ES, requiring correction prior to data collection.

#### How to report it?

The report on logging content inaccuracies should briefly describe the test method, detailing (i) the number of different conditions tested (considering unique combinations of nested conditions), (ii) the number of individual events tested within each condition, and (iii) the count of events that were incorrectly logged out of the total presented. Ideally, in a fully functioning experiment during final data collection, this count of inaccurately logged events should be zero.

### Uncontrolled events

#### Why test it?

Uncontrolled events are those that depend on the participants’ behavior without any control from the experimenter (see Box 1), which are made on devices recorded by the EC (and not on peripherals). This test validates the assumption that the EC correctly registers EC responses into the log files. This is done by comparing the actual responses (made by the experimenter during the test) with the logged ones (recorded in the log files).

#### How to test it?

To validate the fidelity of the logged responses, the experiment should be run from start to end, recording the actual and logged responses made under a systematic, preplanned response sequence. This response plan provides a “recipe” to evaluate whether responses are properly logged and correspond to the executed responses. The purpose of the plan is to test the correct assignment of response buttons, counterbalancing, handling multiple or erroneous responses per single event, and logging responses that occur at unexpected moments during the experiment. To test the correct assignment of response buttons, the response plan should include at least one response of each type the participant is expected to make. When response mapping is counterbalanced within an experiment (e.g., a key is mapped to “Yes” in one block and “No” in another), the response plan should also include responses of the same buttons before and after such changes to determine whether the mappings are reflected in the log files. Handling multiple or erroneous responses is crucial, as participants’ behavior might deviate from that expected by the researchers. Therefore, the response plan should include responses using unexpected keys, cases where a key is pressed more or fewer times than expected (e.g., multiple presses when a single press is expected), or when more than one button is pressed simultaneously.

The final step is to compare the response plan with those recorded on the log file. If executed responses followed the response plan, any incompatibility found points to errors in the ES which require correction prior to data collection.

#### How to report it?

The count of uncontrolled events' content inaccuracies should include a description of the test procedure and response plan, along with three key metrics: (i) the number of different response types (various buttons pressed), (ii) the number of responses for each type, and (iii) the count of responses inaccurately logged out of the total responses. Ideally, in the final data collection phase, the number of inaccurately logged responses should be zero.

## Testing the reliability of the log file event timing

### Controlled events

#### Why test it?

Malfunctions in hardware or software can lead to inaccuracies or a lack of synchrony among three crucial timestamps: the time when a request to display an event is made, the actual occurrence of the event in the experimental hardware, and the time of the event as recorded in the log file. Discrepancies between these timings often reflect EC and ES limitations, rather than human error. Once a request to present an event occurs, the EC processes it along with other requests received at a given time leading to potential delays in the execution. In addition, the computer presents stimuli at a given refresh rate, limiting the display update to a certain number of times per second. As such, the stimulus presentation request has a narrow window to be processed, and when missed, the presentation only occurs in the next frame, unintentionally prolonging the previous event. Various factors (e.g., the EC’s graphics processing unit and CPU, parallel programs running in the background other than the ES) affect processing times and latencies caused by them. Furthermore, logging the timing of an event in the log files is inferred rather than logged in real time, as modern computers do not operate in real time. Thus, discrepancies can arise between the actual event timing and the inferred time recorded in the log file. As such, jitters are to be expected. Yet, large timing deviations require intervention in either the EC or ES. For example, the EC might have insufficient system resources available to run the experiment (solution: free up EC memory, stop unnecessary programs running in the background, e.g., antivirus), or the ES code might be written inefficiently (solution: improve the ES based on the specifics of the software being used). Our simulations showed that inaccuracies in event timing can affect results, which can be detrimental in studies requiring high timing precision (e.g., visual masking paradigms). The significance of precise timing in experiments has been acknowledged before (Plant, 2016), and various solutions to reduce inaccuracies have been proposed (Calcagnotto et al., 2021; Kothe et al., 2024). Experimental environments vary greatly, and so do the patterns of temporal discrepancies across setups (Bridges et al., 2020). Thus, characterizing and reporting the differences between environments is critical for comparing results across studies.

#### How to test it?

To evaluate event timing, a "ground truth" measurement, representing the actual physical timing of events, is essential. This requires an external device like a photodiode, which detects light changes, to record visual events on the screen. By attaching a photodiode to the screen and displaying luminance changes (like black versus white) in sync with the stimulus presentation, one can measure the start and end of each event and calculate its duration. Therefore, to accurately track event timings in an experiment, the experimental software (ES) should be modified to include extreme luminance changes alongside experimental events. The testing process involves the following steps:

##### Modify the ES to allow photodiode-based testing

**Fig. 7:**
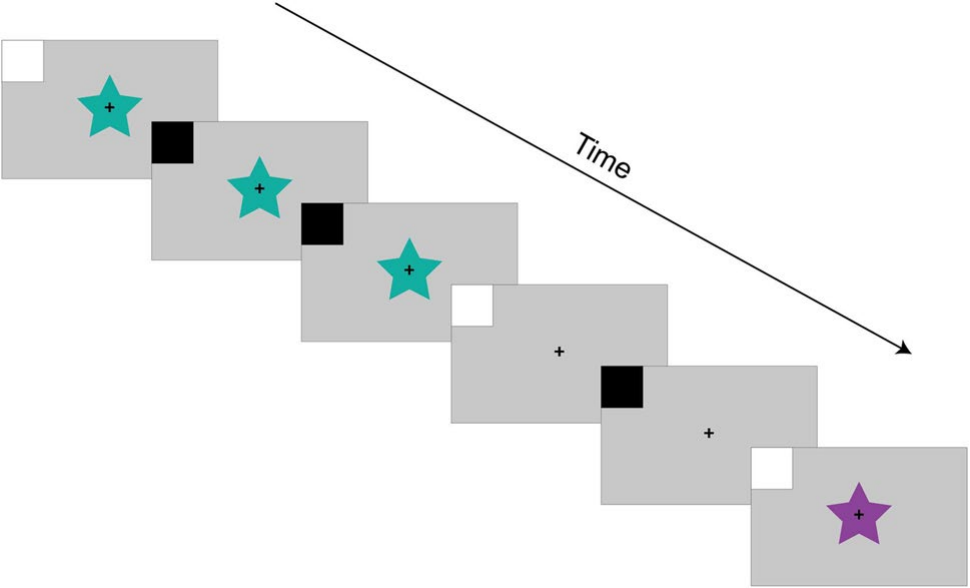
Depiction of visual presentation adjustment to enable testing of timing with a photodiode. A square at the corner of the screen should be flashed to white simultaneously with the onset of each event of interest and return to black thereafter. In this example, the square flashes to white at the onset of each visual stimulus (a colored star) as well as at the offset of each visual stimulus. Critically, the square should not remain white for the entire duration of the stimulus but only for a brief duration at the onset to enable the detection of transitions between each event of interest

While a photodiode sensor can be placed in any location where events appear on the screen, we advocate for a systematic approach. In this step, the researchers integrate into the ES the simultaneous presentations of the evaluated event and a square at one screen corner (or a location not overlapping with the stimulus, where the photodiode is easily attached). The square should “turn on” (e.g., white; RGB 255, 255, 255) at each event onset and offset, and should be “turned off” (e.g., black; RGB 0, 0, 0) otherwise (or vice versa; Fig. 7). This can be achieved by drawing both the test square and the visual event to a back buffer before querying the EC to display a new frame such that both stimulus and test square overlap in time.

##### Attach the photodiode and run the experiment

Once the ES displays the test square, place the photodiode in the location displaying the square, and run the experiment in its entirety. This step will create two files to be compared in the following steps: the log file and the photodiode output file, where the luminance level was recorded.

##### Extract event onsets and offsets from the recorded signal

The next step is to parse the recorded photodiode signal, which is done by setting a threshold discriminating between the two photodiode states, i.e., “on” and “off” (see Fig. 8.1.). The threshold binarizes the signal such that values of “1” indicate samples above threshold (“on”), and “0” sample below threshold (“off”). Then, the onset of each event can be retrieved by finding the transition from off to on samples. This is achieved by computing the discrete difference (i.e., the difference between sample *n*+1 and sample *n* in the signal, see Fig. 8.2 and 7.3) and locating the time points where this difference is equal to 1 (see Fig. 8.4). Importantly, this step should yield the timestamp of the event in temporal units (seconds or milliseconds) by indexing the continuous time vector of the recording. Alternatively, sample units can be converted to seconds by multiplying the sample by the inverse of the sampling frequency.

**Fig. 8:**
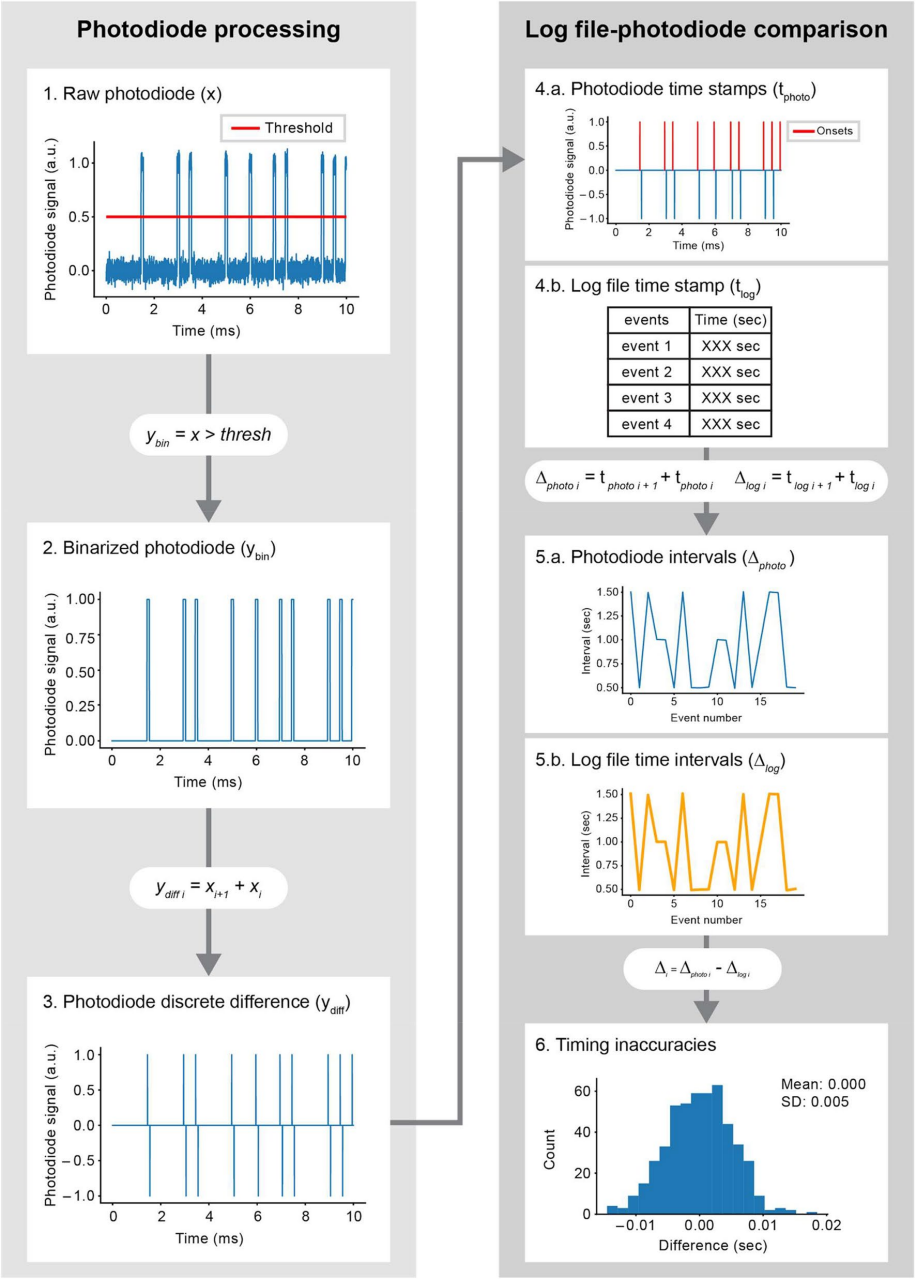
Pipeline to compute the log file timestamping delays using the photodiode. The recorded signal must first be parsed (left panel) to extract the photodiode timestamps. Then, the extracted timestamps can be compared to the log file timestamping by investigating the difference between intervals of successive events as recorded by the photodiode and log file (right panel)

##### Compare photodiode event timings to log files

The initial test ensures that the count of events detected by the photodiode aligns with those logged in the file. If the log file content has already been confirmed, any mismatch in event numbers could indicate issues with the photodiode signal quality. A reliable measuring device (photodiode) is required in order to be considered “ground truth” and is a prerequisite for testing the experiment event timing.

Assuming a reliable photodiode recording, then the photodiode measurements are compared to the log file timings. Two values need to be computed: the discrete difference between successive events’ timestamps in the (1) photodiode (${\Delta }_{photo i}$), and those logged by the (2) EC (${\Delta }_{log i}$):

3 $$\begin{array}{lc}{\Delta }_{log i }= {t}_{log i+1 }- {t}_{log i }\ \ \ \ \ \ \ \ \ \ \ \ \ \ for\ i=1, \dots , n\\ {\Delta }_{photo i }= {t}_{photo i+1 }- {t}_{photo i }\ \ \ \ for\ i=1, \dots , n\end{array}$$

where ${t}_{photo i}$ and ${t}_{log i}$ are the timestamps for a given $i$ event in the photodiode recording and the experimental output, respectively (see Fig. 8.5.a and b). The logging timestamping inaccuracy is then computed as the difference between ${\Delta }_{photo i}$ and ${\Delta }_{log i}$ as

4 $${\Delta }_{i}={\Delta }_{photo i }-{\Delta }_{log i }\ \ \ \ for i=1, \dots , n$$

${\Delta }_{i}$ constitutes the log file timestamping inaccuracy for every single event (see Fig. 8.6), assuming a reliable photodiode.

For a well-calibrated setup, the expected difference between ${\Delta }_{photo i}$ and ${\Delta }_{log i}$ is on the order of milliseconds or lower (Bridges et al., 2020). The average in ${\Delta }_{i}$ should approximate zero, with a small standard deviation in the order of a few milliseconds. Large discrepancies indicate a problem, either with the timing at which events are displayed or at which they are logged. Inspecting the difference between both timestamp vectors might reveal the cause of those discrepancies (missing events, events that are systematically displaced in time, etc.).

#### How to report it?

The average and standard deviation values of ${\Delta }_{i}$ across events should be reported.

## Uncontrolled events

### Why test it?

Uncontrolled event timing (e.g., reaction times) can also be affected by hardware and software limitations. Reaction time effects are often in the range of tens of milliseconds (Schlossmacher et al., 2020; van Gaal et al., 2010), making it necessary to determine the recording precision. Timestamping of uncontrolled events can show *delays* as well as *jitters*. It is therefore crucial to test both and report the results.

#### How to test it?

A method advocated by Psychtoolbox (and their KeyboardLatencyTest method) is to concurrently record the sound associated with the actual press of a button. This is done by placing a microphone close to the response device used in the experiment. This requires a modification of the ES to log the microphone-recorded sound into a file. Then, the button press onset can be extracted from the audio file and compared to the timestamps of responses recorded in the log file. The steps are as follows:

##### Modify the ES to record sound

This can be done by adding a statement at the beginning of the code to continuously record the sound throughout the experiment.

##### Attach the microphone and run the experiment

A contact microphone should be attached close to the keys being pressed on the response device. Sharply pressing the keys during the test run ensures easy processing in the next step. External recording devices (photodiode, microphone) are for testing purposes only and can be removed for data collection.

After obtaining the microphone recordings and log files, the analysis steps to extract and compare the triggers are the same as the procedure described in the sections “Extract event onsets and offsets from the recorded signal” and “*Compare photodiode event timings to log files*,” to compute the ${\Delta }_{r}$ (Eq. 4, with *r* denoting responses), respectively.

#### How to report it?

Like the test for the precision of controlled event timing, the average and standard deviation of ${\Delta }_{r}$ should be documented. In an optimally calibrated experimental setting, the average ${\Delta }_{r}$ is expected to be near zero, with the standard deviation within the range of milliseconds.

## Validating the experimental design parameters

### Why test it?

After the content and timing of events has been validated, specific aspects of the experimental design can be evaluated (e.g., the duration and balancing of event groups, conditions, their sequential presentation). As there are countless choices of experimental designs, our aim is not to cover all potential designs, but instead to provide the community with a systematic method for testing and reporting experimental design aspects.

#### How to test it?

Below, we focus on two examples validating the experimental design with respect to content and timing.

Concerning adhering to the experimental design content, the aim is to confirm that those rules that researchers wish to enforce in the controlled events (e.g., presented stimuli) are indeed implemented (e.g., order of presentation, constraints on sequential trials). To test the content requirements, the following steps are proposed:

##### Know the experiment content requirements

Document as explicitly as possible what is expected to be enforced, e.g., number of stimulus repetitions, randomization scheme, event order, stimulus locations, and balancing of event groups. Every requirement pertaining to event content in the experimental design should be specified and checked.

##### Ensure that the relevant information is recorded in the log file

Ensure the information required to test the previous step is stored in the log file.

##### Prepare checks to make sure that the log file meets each requirement

Prepare, ideally, a programmatic script that reads the log file and ensures that its content adheres to each requirement listed in the first step following the completion of the test run. In cases where researchers have pre-made sequences including all the information about the flow of the events within an experiment, tests can be conducted in those pre-made files.

With respect to validating the timings of the experimental design, the goal is to assess how closely the actual durations of events align with their intended durations. Here, after comparing the photodiode output with the log file, researchers compare the actual duration of the observed events with their planned duration, as specified in the experimental design timing scheme.

The first step is to obtain the observed event durations from the photodiode recordings:

5 $${Obs\;Duration}_{i}=\;{OEO}_{i+1 }-\;{OEO}_{i} for i=1, \dots , n$$

where OEO stands for the onset and offset of each event i. Then, one can compute how much these durations deviate from the plan:

6 $${\Delta }_{i }= {Obs\;Duration}_{i}-{Planned\;duration}_{i}\;fori=1, \dots , n$$

where $Planned duration$ is the planned duration of each event $i$ according to the experimental design.

#### How to report it?

The report for experiments with varying experimental design content-based rules should include a comprehensive list of design choices. Evaluating how well the design meets these requirements involves counting the number of events, out of the total tested, that comply with each requirement. For nested designs, report the number of events for each combination. If relevant, include the count of events per condition in each block. Notably, there should be no deviations, as any would suggest noncompliance with the study's plan. If content errors are discovered, a reassessment of the experimental software (ES) is needed, followed by retesting after corrections are made and inaccuracies are corrected.

For event timing, the precision of the planned timings should be reported. We advocate reporting the mean and standard deviation ${\Delta }_{i}$. The mean is expected to approach zero, and the standard deviation to be within the few milliseconds range. Larger values are suggestive of errors or hardware issues that might require attention.

## Testing peripheral triggers

### Why test it?

So far, we have described tests to benchmark the EC and ES. Yet, when the experimental environment contains peripherals (a typical case for neuroscience experiments), tests to assess the interaction of the peripherals with the EC and ES (i.e., triggers) are also necessary. Triggers serve a dual purpose here: (1) they provide temporal markers for events of interest on which to focus offline data analysis, and (2) they play a pivotal role in maintaining synchrony between the EC and the recording system (e.g., electroencephalography [EEG], eye-tracking device). This is useful for addressing issues related to clock drift, which can be detected and corrected when sending triggers marking events of interest to multiple devices (Niso et al., 2022).

The interpretation of the signal recorded by these devices depends on the integrity of the trigger transmissions (Boudewyn et al., 2023; Luck, 2014), which is the focus of the current test. Akin to previous tests, both the content and the timing of the triggers representing the controlled and uncontrolled events are evaluated. These tests should be performed for each peripheral device used in the experiment.

#### How to test it?

##### Peripheral trigger content

Assuming the log file event records are accurate, the congruence between each logged event and its corresponding trigger content is assessed. Any deviations point to problems with trigger logging or the peripheral device, necessitating review and correction.

##### Peripheral trigger timing

Compute the discrete difference between successive peripheral trigger timestamps, and compare it against the discrete differences between consecutive observed events, as recorded by the photodiode. The difference between these two arrays provides an estimate of the peripheral trigger temporal jitter (${\Delta }_{i}$):

7 $${\Delta }_{i }=\left({t}_{photo i+1 }- {t}_{photo i}\right)-\left({t}_{trig i+1 }- {t}_{trig i}\right) for\;i=1, \dots , n$$

Note that this method evaluates temporal *jitter* but is not suited to evaluate *delays* between events and peripheral triggers. As described by Farzan et al. (2017), delays can be measured by recording the photodiode signal on the same computer clock as the peripheral of interest. The delay of the trigger onset (marked below as ${\Delta }_{i}$ too) can then be directly compared to the detected photodiode onset as:

8 $${\Delta }_{i }= {t}_{photo i}- {t}_{trig i}\ \ \ \ \ for\ i=1, \dots , n$$

#### How to report it?

##### Peripheral trigger content

The count of events where the trigger content does not align with the log file content should be documented, and reported as the count of mismatched events over the total count of events.

##### Peripheral trigger timing

The report should contain the average and standard deviation of trigger timings. For systematic delay tests, both the mean and standard deviation should be reported.

## Standardized report

The framework is summarized in a checklist available on protocols.io, alongside a standardized format for reporting these results. Both the checklist and the report are in the protocols.io platform, under this link. Testing the experimental environment is crucial, and so is the accompanying detailed report of the test results to altogether enhance transparency and foster reproducibility and replicability.

## Discussion

We present a framework to systematically test and report the performance of experimental environments. We aimed to minimize financial burden by relying on hardware that either most labs already have, or is inexpensive to acquire or build (e.g., photodiode and microphone). Our protocol enables all tests to be conducted in a single test run, making the framework more efficient for researchers and preventing additional work due to errors or limitations discovered during the data collection stage. Furthermore, the framework also includes guidelines for detailed reporting of the parameters and results of each test, as means to increase scientific transparency. Such transparency allows the scientific community to better evaluate the conclusions of the study, as they rely heavily on the proper functioning of the experiment. As indicated by the survey results, more than half of researchers did not share their test results because they did not know where or how to write them; the current framework would hopefully help overcome this hurdle.

The framework presented herein is designed to be applicable to most recording modalities used in human neurosciences. To test the content and timing of the experimental events, the only requirement is that the peripherals are capable of receiving triggers (which is a standard feature in experimental environments containing peripherals in event-related designs). To assess event timing accuracy, jitters are estimated by comparing the intervals between events recorded by the system with those obtained from physical measurements: jitter-free peripherals should show identical intervals. Although the clocks of different devices may drift apart over long periods, computing the intervals between events occurring in relatively short succession overcomes this problem. Notably, this method does not account for systematic delays between two systems (i.e., if a peripheral device receives the triggers with a systematic delay of 30 ms with respect to the physical event). To address this, systematic delays should be quantified by recording the physical signals on the same computer as the peripheral being used, as described in section "Testing peripheral triggers" (Eq. 8). As such, our framework offers a comprehensive set of tests for timing issues applicable to a wide range of technologies used in cognitive neuroscience.

Investing extra time and resources in testing and reporting the experimental environment is worthwhile, as simulations show that malfunctions in recording event timing and content can significantly impact results. Researchers should aim to reduce controllable errors and characterize noise in their setups to increase the likelihood of detecting real effects and reduce false negatives. The benefits of this framework extend beyond individual experiments, enhancing replication efforts and scientific reliability. Recent replication challenges in neuroscience and psychology (e.g., Kristal et al., 2020) highlight the need for quality assurance in experimental environments, especially when multiple labs collaborate (e.g., COGITATE: COGITATE Consortium et al., 2023; eegManyLabs: Pavlov et al., 2021; The International Brain Laboratory et al., 2021). Without strict quality controls, the potential benefits of multi-site data collection risk being overshadowed by inter-site variability, masking real effects observable in single-site datasets (e.g., de Vries et al., 2022; Farzan et al., 2017). The step-by-step process of conducting the framework on protocols.io enables researchers to thoroughly test their experiments in an effective and relatively non-time-consuming manner. A detailed demonstration of the application of our framework to an experiment is provided as a Jupyter notebook (see Supplemental Material 3).

We believe the short time invested is outweighed by the benefits in the long run. We acknowledge that for some, the proposed framework and reporting approach might seem excessive and may also be met with skepticism, as it may increase the burden on the researchers. Yet, as our survey shows, most researchers do encounter issues only after data collection begins. Addressing errors retrospectively is time-consuming, and in extreme cases, undetected issues can lead to retractions (e.g., Grave et al., 2021) or flawed results. Standardized testing and reporting can identify problems early, aiding replication and consistency across studies, including multi-lab projects. We hope this practice will gradually become integral to good scientific conduct, similar to the adoption of preregistration, which was initially met with skepticism (Paret et al., 2022), as it required more effort and resources—but over time proved to be highly beneficial (Gentili et al., 2021; Protzko et al., 2023).

Finally, we argue that our proposed framework may not be costlier than current testing practices in the field. Our survey indicates that researchers already invest time in testing their experimental setups and also recognize the need for testing before data collection. Yet, these efforts often go unreported. Without a standardized test protocol, each researcher and lab must devise their own methods, with many creating unique tests for each experiment. Our framework outlines four key aspects to test in event-based experiments: reliability of log file (1) event content and (2) event timing, (3) fulfillment of the experimental design, and (4) reliability of the peripheral device. These tests are broad enough to cover most visual presentation designs, needing only minor adjustments for specific cases. With careful planning, a single full experimental run, including all peripherals, can suffice for comprehensive testing. Most steps, except for logging event content, can be automated with minimal execution time. While script development might initially take time, these scripts are generally reusable across studies, offering long-term efficiency.

Thus, we believe that the research community can benefit from these resources. This framework can enhance the credibility of research findings, improve research efficiency and cost-effectiveness, and, by reporting test results, increase the transparency and reproducibility of research methods.

## Supplementary Information

Below is the link to the electronic supplementary material.

Supplementary file1 (DOCX 1588 KB)

## Data Availability

All code and data used and generated for this paper are openly available at https://github.com/Cogitate-consortium/ExperimentTestingFramework

## References

[CR1] Baxter, M. G., & Burwell, R. D. (2017). Promoting transparency and reproducibility in Behavioral Neuroscience: Publishing replications, registered reports, and null results. *Behavioral Neuroscience,**131*(4), 275–276. 10.1037/bne0000207 28714713 10.1037/bne0000207 PMC6102714

[CR2] Boudewyn, M. A., Erickson, M. A., Winsler, K., Ragland, J. D., Yonelinas, A., Frank, M., Silverstein, S. M., Gold, J., MacDonald III, A. W., Carter, C. S., Barch, D. M., & Luck, S. J. (2023). Managing EEG studies: How to prepare and what to do once data collection has begun. *Psychophysiology*, *n/a*(n/a), e14365. 10.1111/psyp.14365 10.1111/psyp.14365 PMC11276027 37314113

[CR3] Brainard, D. H. (1997). The psychophysics toolbox. *Spatial Vision,**10*(4), 433–436. 9176952

[CR4] Bridges, D., Pitiot, A., MacAskill, M. R., & Peirce, J. W. (2020). The timing mega-study: Comparing a range of experiment generators, both lab-based and online. *PeerJ,**8*, e9414. 10.7717/peerj.9414 33005482 10.7717/peerj.9414 PMC7512138

[CR5] Button, K. S., Ioannidis, J. P., Mokrysz, C., Nosek, B. A., Flint, J., Robinson, E. S., & Munafò, M. R. (2013). Power failure: why small sample size undermines the reliability of neuroscience. *Nature Reviews Neuroscience,**14*(5), 365–376. 23571845 10.1038/nrn3475

[CR6] Calcagnotto, L., Huskey, R., & Kosicki, G. M. (2021). The accuracy and precision of measurement: Tools for validating reaction time stimuli. *Computational Communication Research,**3*(2), 1–20.

[CR7] Carp, J. (2012). On the Plurality of (Methodological) Worlds: Estimating the Analytic Flexibility of fMRI Experiments. *Frontiers in Neuroscience,**6*, 149. 10.3389/fnins.2012.00149 23087605 10.3389/fnins.2012.00149 PMC3468892

[CR8] Consortium, C., Ferrante, O., Gorska-Klimowska, U., Henin, S., Hirschhorn, R., Khalaf, A., Lepauvre, A., Liu, L., Richter, D., Vidal, Y., Bonacchi, N., Brown, T., Sripad, P., Armendariz, M., Bendtz, K., Ghafari, T., Hetenyi, D., Jeschke, J., Kozma, C., …, & Melloni, L. (2023). *An adversarial collaboration to critically evaluate theories of consciousness* (p. 2023.06.23.546249). bioRxiv. 10.1101/2023.06.23.546249

[CR9] Cumming, G. (2014). The new statistics: why and how. *Psychological Science,**25*(1), 7–29. 24220629 10.1177/0956797613504966

[CR10] de Vries, S. E. J., Siegle, J. H., & Koch, C. (2022). *Sharing Neurophysiology Data from the Allen Brain Observatory: Lessons Learned* (arXiv:2212.08638). arXiv. 10.48550/arXiv.2212.08638 10.7554/eLife.85550 PMC10335829 37432073

[CR11] Fanelli, D. (2012). Negative results are disappearing from most disciplines and countries. *Scientometrics,**90*(3), 891–904.

[CR12] Farzan, F., Atluri, S., Frehlich, M., Dhami, P., Kleffner, K., Price, R., Lam, R. W., Frey, B. N., Milev, R., Ravindran, A., McAndrews, M. P., Wong, W., Blumberger, D., Daskalakis, Z. J., Vila-Rodriguez, F., Alonso, E., Brenner, C. A., Liotti, M., Dharsee, M., & Kennedy, S. H. (2017). Standardization of electroencephalography for multi-site, multi-platform and multi-investigator studies: Insights from the Canadian biomarker integration network in depression. *Scientific Reports,**7*(1), 1. 10.1038/s41598-017-07613-x 28785082 10.1038/s41598-017-07613-x PMC5547036

[CR13] Frank, M. C., Bergelson, E., Bergmann, C., Cristia, A., Floccia, C., Gervain, J., Hamlin, J. K., Hannon, E. E., Kline, M., Levelt, C., Lew-Williams, C., Nazzi, T., Panneton, R., Rabagliati, H., Soderstrom, M., Sullivan, J., Waxman, S., & Yurovsky, D. (2017). A Collaborative approach to infant research: Promoting reproducibility, best practices, and theory-building. *Infancy,**22*(4), 421–435. 10.1111/infa.12182 31772509 10.1111/infa.12182 PMC6879177

[CR14] Gentili, C., Cecchetti, L., Handjaras, G., Lettieri, G., & Cristea, I. A. (2021). The case for preregistering all region of interest (ROI) analyses in neuroimaging research. *European Journal of Neuroscience,**53*(2), 357–361. 10.1111/ejn.14954 32852863 10.1111/ejn.14954

[CR15] Grave, J., Soares, S. C., Morais, S., Rodrigues, P., & Madeira, N. (2021). Retraction notice to “The effects of perceptual load in processing emotional facial expression in psychotic disorders” [Psychiatry Research Volume 250C April 2017, pages 121—128]. *Psychiatry Research,**303*, 114077. 10.1016/j.psychres.2021.114077 34247059 10.1016/j.psychres.2021.114077

[CR16] Hirschhorn, R., & Schonberg, T. (2024). Replication. In *Encyclopedia of the Human Brain* (2nd ed.). Elsevier.

[CR17] Ioannidis, J. P. (2005). Why most published research findings are false. *PLoS Medicine,**2*(8), e124. 10.1371/journal.pmed.0020124 PMC1182327 16060722

[CR18] Kothe, C., Shirazi, S. Y., Stenner, T., Medine, D., Boulay, C., Crivich, M. I., ... & Makeig, S. (2024). The lab streaming layer for synchronized multimodal recording. *bioRxiv*, 2024-02. 10.1101/2024.02.13.580071 10.1162/IMAG.a.136 PMC12434378 40959706

[CR19] Kristal, A. S., Whillans, A. V., Bazerman, M. H., Gino, F., Shu, L. L., Mazar, N., & Ariely, D. (2020). Signing at the beginning versus at the end does not decrease dishonesty. *Proceedings of the National Academy of Sciences,**117*(13), 7103–7107. 10.1073/pnas.1911695117 10.1073/pnas.1911695117 PMC7132248 32179683

[CR20] Logg, J. M., & Dorison, C. A. (2021). Pre-registration: Weighing costs and benefits for researchers. *Organizational Behavior and Human Decision Processes,**167*, 18–27. 10.1016/j.obhdp.2021.05.006

[CR21] Luck, S. J. (2014). *An Introduction to the Event-Related Potential Technique* (2nd ed.). MIT Press.

[CR22] Melloni, L., Mudrik, L., Pitts, M., & Koch, C. (2021). Making the hard problem of consciousness easier. *Science,**372*(6545), 911–912. 10.1126/science.abj3259 34045342 10.1126/science.abj3259

[CR23] Mumford, J. A., & Nichols, T. E. (2008). Power calculation for group fMRI studies accounting for arbitrary design and temporal autocorrelation. *NeuroImage,**39*(1), 261–268. 10.1016/j.neuroimage.2007.07.061 17919925 10.1016/j.neuroimage.2007.07.061 PMC2423281

[CR24] Munafò, M. R., Nosek, B. A., Bishop, D. V., Button, K. S., Chambers, C. D., Du Sert, N. P., & Ioannidis, J. P. (2017). A manifesto for reproducible science. *Nature Human Behaviour,**1*(1), 1–9. 10.1038/s41562-016-0021 10.1038/s41562-016-0021 PMC7610724 33954258

[CR25] Niso, G., Krol, L. R., Combrisson, E., Dubarry, A. S., Elliott, M. A., François, C., Héjja-Brichard, Y., Herbst, S. K., Jerbi, K., Kovic, V., Lehongre, K., Luck, S. J., Mercier, M., Mosher, J. C., Pavlov, Y. G., Puce, A., Schettino, A., Schön, D., Sinnott-Armstrong, W., …, Chaumon, M. (2022). Good scientific practice in EEG and MEG research: Progress and perspectives. *NeuroImage*, *257*, 119056. 10.1016/j.neuroimage.2022.119056 10.1016/j.neuroimage.2022.119056 PMC11236277 35283287

[CR26] Open Science Collaboration. (2015). Estimating the reproducibility of psychological science. *Science,**349*(6251), aac4716. 10.1126/science.aac4716 26315443

[CR27] Paret, C., Unverhau, N., Feingold, F., Poldrack, R. A., Stirner, M., Schmahl, C., & Sicorello, M. (2022). Survey on open science practices in functional neuroimaging. *NeuroImage,**257*, 119306. 10.1016/j.neuroimage.2022.119306 35595201 10.1016/j.neuroimage.2022.119306

[CR28] Pavlov, Y. G., Adamian, N., Appelhoff, S., Arvaneh, M., Benwell, C. S. Y., Beste, C., Bland, A. R., Bradford, D. E., Bublatzky, F., Busch, N. A., Clayson, P. E., Cruse, D., Czeszumski, A., Dreber, A., Dumas, G., Ehinger, B., Ganis, G., He, X., Hinojosa, J. A., …, & Mushtaq, F. (2021). #EEGManyLabs: Investigating the replicability of influential EEG experiments. *Cortex*, *144*, 213–229. 10.1016/j.cortex.2021.03.013 10.1016/j.cortex.2021.03.013 33965167

[CR29] Peirce, J. W. (2007). PsychoPy—Psychophysics software in Python. *Journal of Neuroscience Methods,**162*(1), 8–13. 10.1016/j.jneumeth.2006.11.017 17254636 10.1016/j.jneumeth.2006.11.017 PMC2018741

[CR30] Pernet, C., Garrido, M., Gramfort, A., Maurits, N., Michel, C. M., Pang, E., Salmelin, R., Schoffelen, J. M., Valdes-Sosa, P. A., & Puce, A. (2018). *Best practices in data analysis and sharing in neuroimaging using MEEG*. 10.31219/osf.io/a8dhx

[CR31] Plant, R. R. (2016). A reminder on millisecond timing accuracy and potential replication failure in computer-based psychology experiments: An open letter. *Behavior Research Methods,**48*(1), 408–411. 25761394 10.3758/s13428-015-0577-0

[CR32] Poldrack, R. A., Fletcher, P. C., Henson, R. N., Worsley, K. J., Brett, M., & Nichols, T. E. (2008). Guidelines for reporting an fMRI study. *Neuroimage,**40*(2), 409–414. 18191585 10.1016/j.neuroimage.2007.11.048 PMC2287206

[CR33] Protzko, J., Krosnick, J., Nelson, L., Nosek, B. A., Axt, J., Berent, M., Buttrick, N., DeBell, M., Ebersole, C. R., Lundmark, S., MacInnis, B., O’Donnell, M., Perfecto, H., Pustejovsky, J. E., Roeder, S. S., Walleczek, J., & Schooler, J. W. (2023). High replicability of newly discovered social-behavioural findings is achievable. *Nature Human Behaviour,**8*(2), 311–319. 10.1038/s41562-023-01749-9 37945809 10.1038/s41562-023-01749-9 PMC10896719

[CR34] Schlossmacher, I., Dellert, T., Pitts, M., Bruchmann, M., & Straube, T. (2020). Differential Effects of Awareness and Task Relevance on Early and Late ERPs in a No-Report Visual Oddball Paradigm. *Journal of Neuroscience,**40*(14), 2906–2913. 10.1523/JNEUROSCI.2077-19.2020 32122954 10.1523/JNEUROSCI.2077-19.2020 PMC7117899

[CR35] Sejnowski, T. J., Churchland, P. S., & Movshon, J. A. (2014). Putting big data to good use in neuroscience. *Nature Neuroscience,**17*(11), 11. 10.1038/nn.3839 10.1038/nn.3839 PMC4224030 25349909

[CR36] Simmons, J. P., Nelson, L. D., & Simonsohn, U. (2011). False-positive psychology: Undisclosed flexibility in data collection and analysis allows presenting anything as significant. *Psychological Science,**22*(11), 1359–1366. 22006061 10.1177/0956797611417632

[CR37] The International Brain Laboratory, Aguillon-Rodriguez, V., Angelaki, D., Bayer, H., Bonacchi, N., Carandini, M., Cazettes, F., Chapuis, G., Churchland, A. K., Dan, Y., Dewitt, E., Faulkner, M., Forrest, H., Haetzel, L., Häusser, M., Hofer, S. B., Hu, F., Khanal, A., Krasniak, C., …, & Zador, A. M. (2021). Standardized and reproducible measurement of decision-making in mice. *eLife*, *10*, e63711. 10.7554/eLife.63711 10.7554/eLife.63711 PMC8137147 34011433

[CR38] van Gaal, S., Ridderinkhof, K. R., Scholte, H. S., & Lamme, V. A. F. (2010). Unconscious Activation of the Prefrontal No-Go Network. *Journal of Neuroscience,**30*(11), 4143–4150. 10.1523/JNEUROSCI.2992-09.2010 20237284 10.1523/JNEUROSCI.2992-09.2010 PMC6632275

[CR39] Wicherts, J. M., Veldkamp, C. L., Augusteijn, H. E., Bakker, M., Van Aert, R., & Van Assen, M. A. (2016). Degrees of freedom in planning, running, analyzing, and reporting psychological studies: A checklist to avoid p-hacking. *Frontiers in psychology,**7*, 1832. 10.3389/fpsyg.2016.01832 27933012 10.3389/fpsyg.2016.01832 PMC5122713

