# Supplementary material for: A standardized framework to test event-based experiments
Source: Behav Res Methods. 2024 Sep 16;56(8):8852–68. doi: 10.3758/s13428-024-02508-y (PMC11525435; doi:10.3758/s13428-024-02508-y)
Supplement: Supplementary file 1 — Supplementary file1 (DOCX 1588 KB) [file 13428_2024_2508_MOESM1_ESM.docx]

# Supplementary Materials

## 1. Online Survey

One hundred participants took part in a survey aimed at researchers conducting computer-controlled experiments, after providing explicit informed consent, under the approval of the ethics council of the Max Planck society (2017_12). The survey was run on LimeSurvey. Data was collected between January and February 2022. Data collection was anonymized, and no identifiable information was registered. To reach out to a diverse set of researchers, the survey was advertised through social media platforms (e.g., Twitter, Slack) and mailing lists such as SPM, FreeSurfer, MNE, and EEGLab. Before enrolling in the survey, participants were informed about its purpose and the terms of enrollment (e.g., that data were anonymized and the expected duration). After providing explicit consent, individuals were presented with the instructions and completed 7-18 multiple-choice questions (the number of questions depended on the responses, as Section B of the questionnaire was only given to respondents reporting testing their experiment in some way). The complete survey is presented below. In short, the survey included questions about the participants’ practices when testing the experimental environment. It was clarified that “testing” referred to performing tests on the experimental environment prior to beginning data collection. When reporting those, participants were instructed to respond based on the last published experiment they executed themselves.

Below are the questions as they were presented in the online survey. Note that participants who replied” Never” in question A2 were not presented with Section B, and replied only to Section C questions.

### Survey Replies

#### Section A: Initial questions

1. How many different experiments have you carried out (i.e., programmed, ran, analyzed, or a combination of those)?


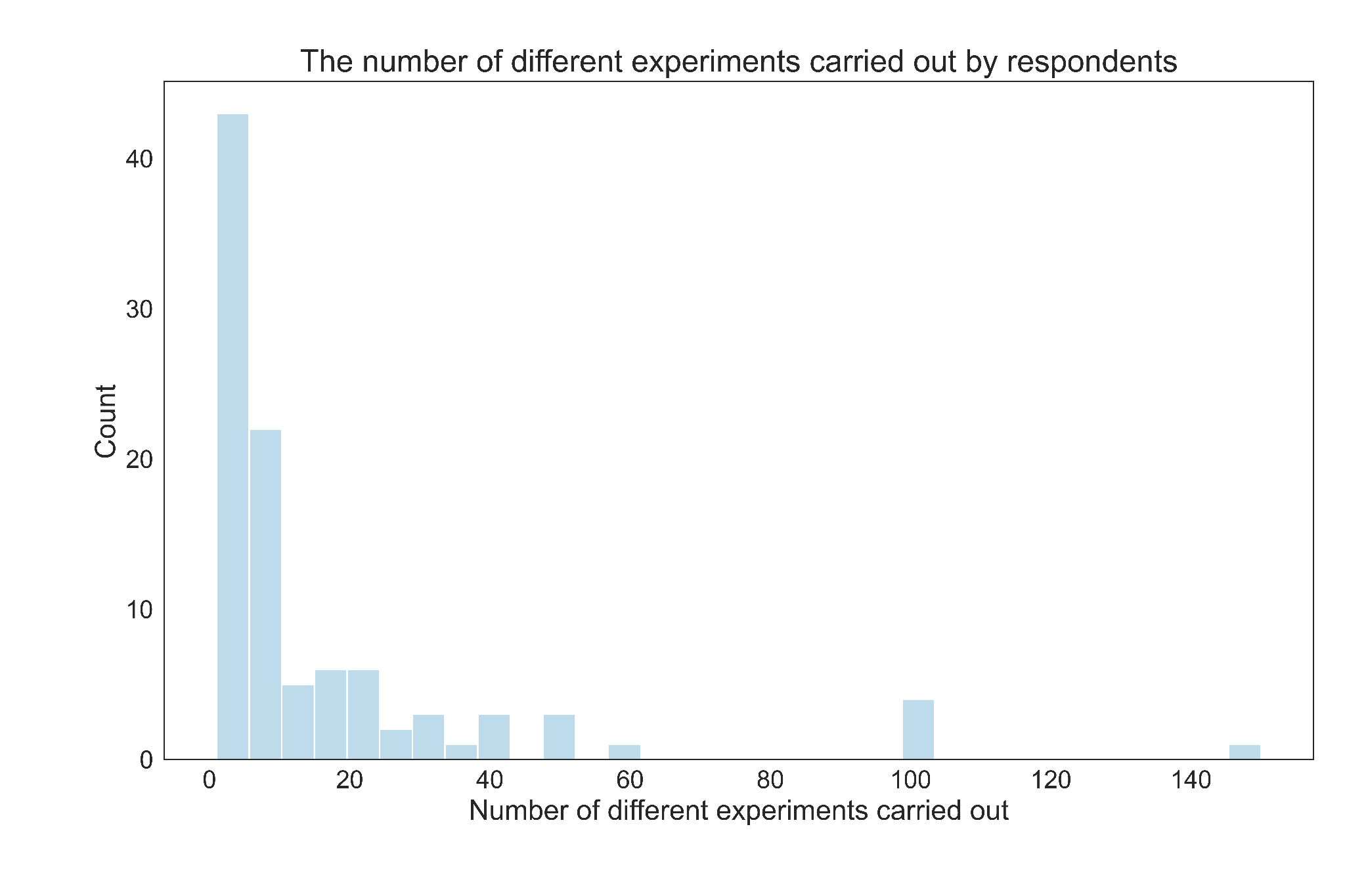


*Figure 1: Histogram of reported number of experiment carried out by survey participantsf*

1. For how many of these experiments did you run the experiment before piloting, with the sole goal of testing that the experiment behaves properly? (i.e., running the way you expect it to, logging the way you expect it to, etc.)
   1. All (100%)
   2. Most (>=50%)
   3. Some (<50%)
   4. Never (0%)


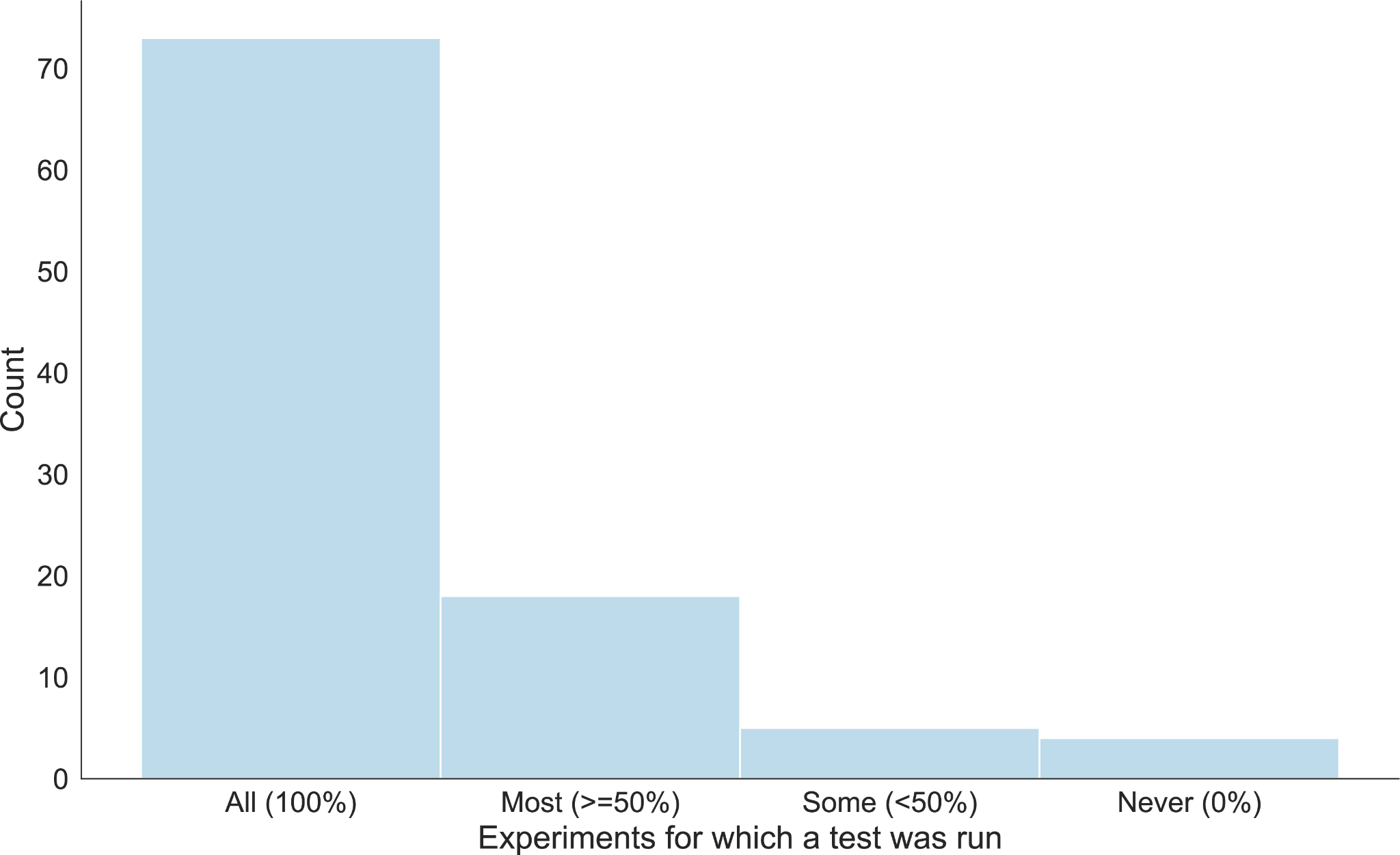


*Figure 2: Histogram of reported proportion of experiment for which a test was run*

#### Section B: Pre-acquisition tests

In the following section, all questions refer to the last published experiment that you carried out!

1. In the last published experiment you carried out, which of the following aspects did you test? (Feel free to add more in the "other" section)
   1. Overall experiment duration
   2. A match between the recorded timing of events and their occurrence on the screen
   3. Content of events (i.e., a match between the content that was presented on the screen and the one that was recorded)
   4. Integration of peripheral devices (e.g., that the connection between a response device and the experiment's software works as expected)
   5. Fulfillment of the pseudo-randomization scheme (i.e., that the constraints on the order of events are respected)
   6. Other [free text]


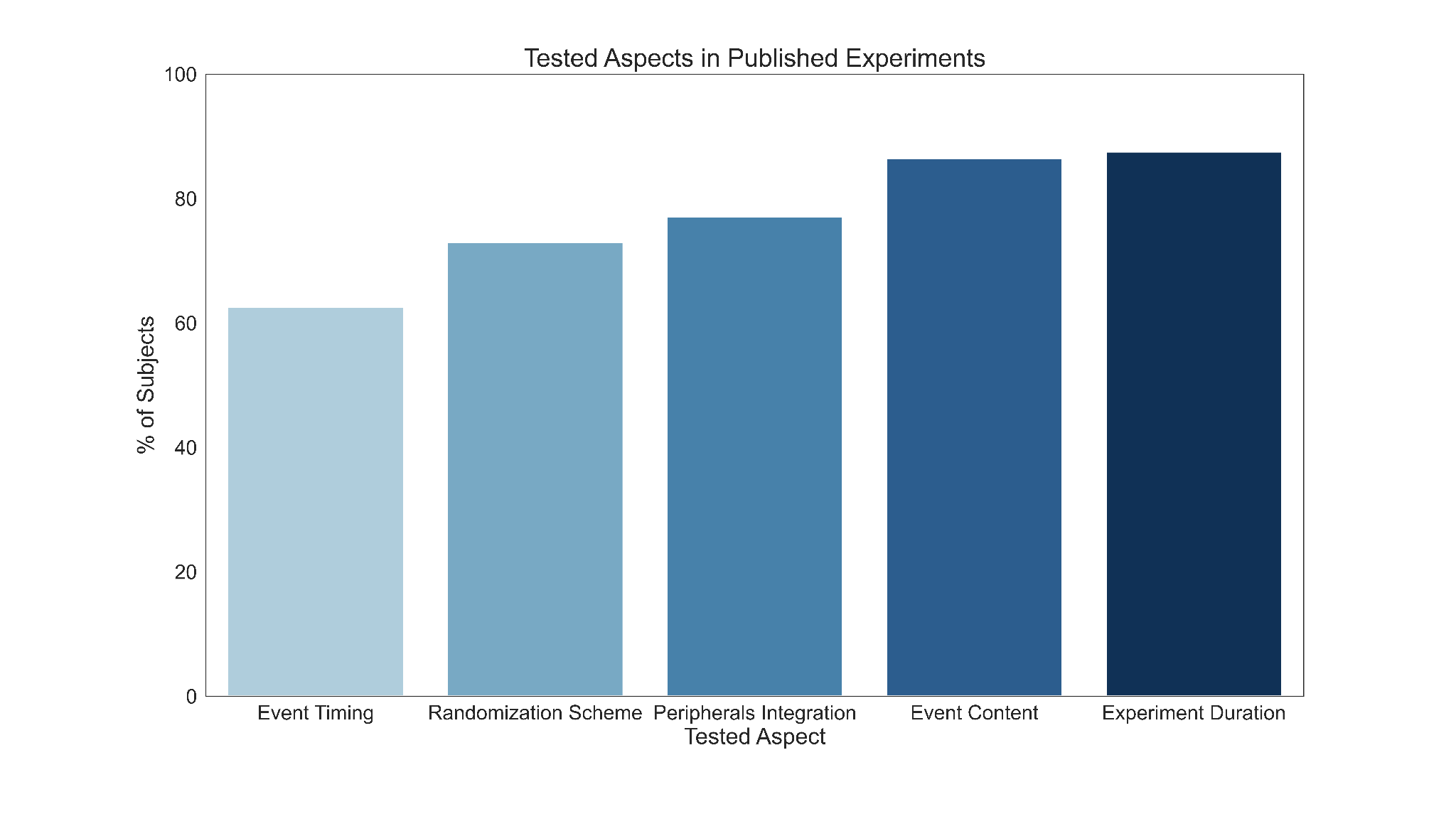


*Figure 3: Histogram of the proportion of survery participants reporting testing a particular aspect of their experiment*

1. Which methods did you use to test the above mentioned parameters? [can check more than one]
   1. Manual checks: looking at the experiment while it's running, going through the outputs of the experiment manually to make sure they make sense
   2. Scripted checks: writing scripts to systematically test certain parameters of the experiment behave as expected


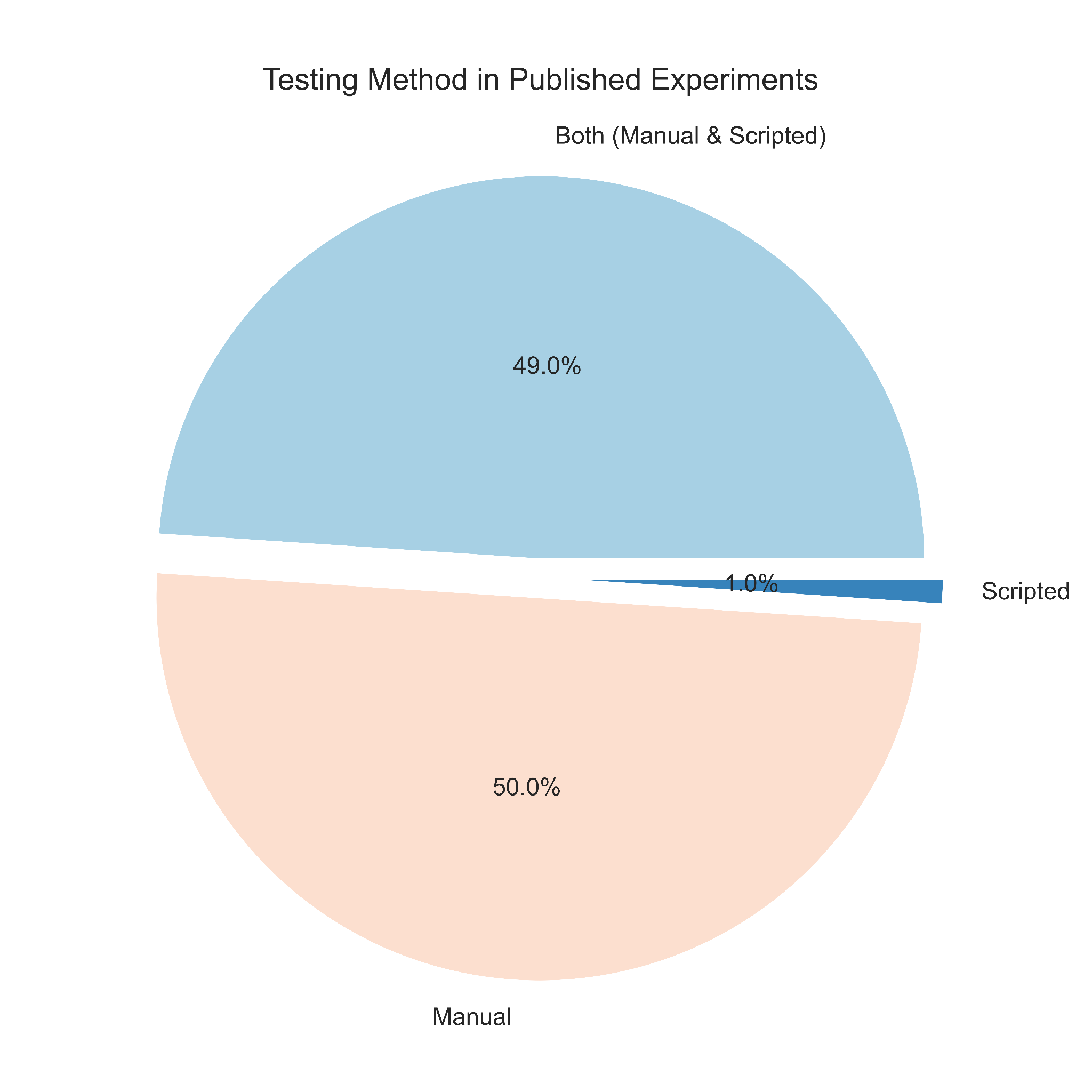


*Figure 4: Pie chart of the proportion of subjects reporting use of manual, scripted or both tests to ensure the well-functioning of their experiment*

1. Were the tests you performed on this specific experimental setup based on any existing testing protocol?
   1. A: I have a protocol of tests all my experiments go through
   2. B: Every experiment had its own protocol of tests
   3. I used to follow A, now I do B
   4. I used to follow B, not I do A
   5. I do a mixture of A and B
   6. Other [free text]


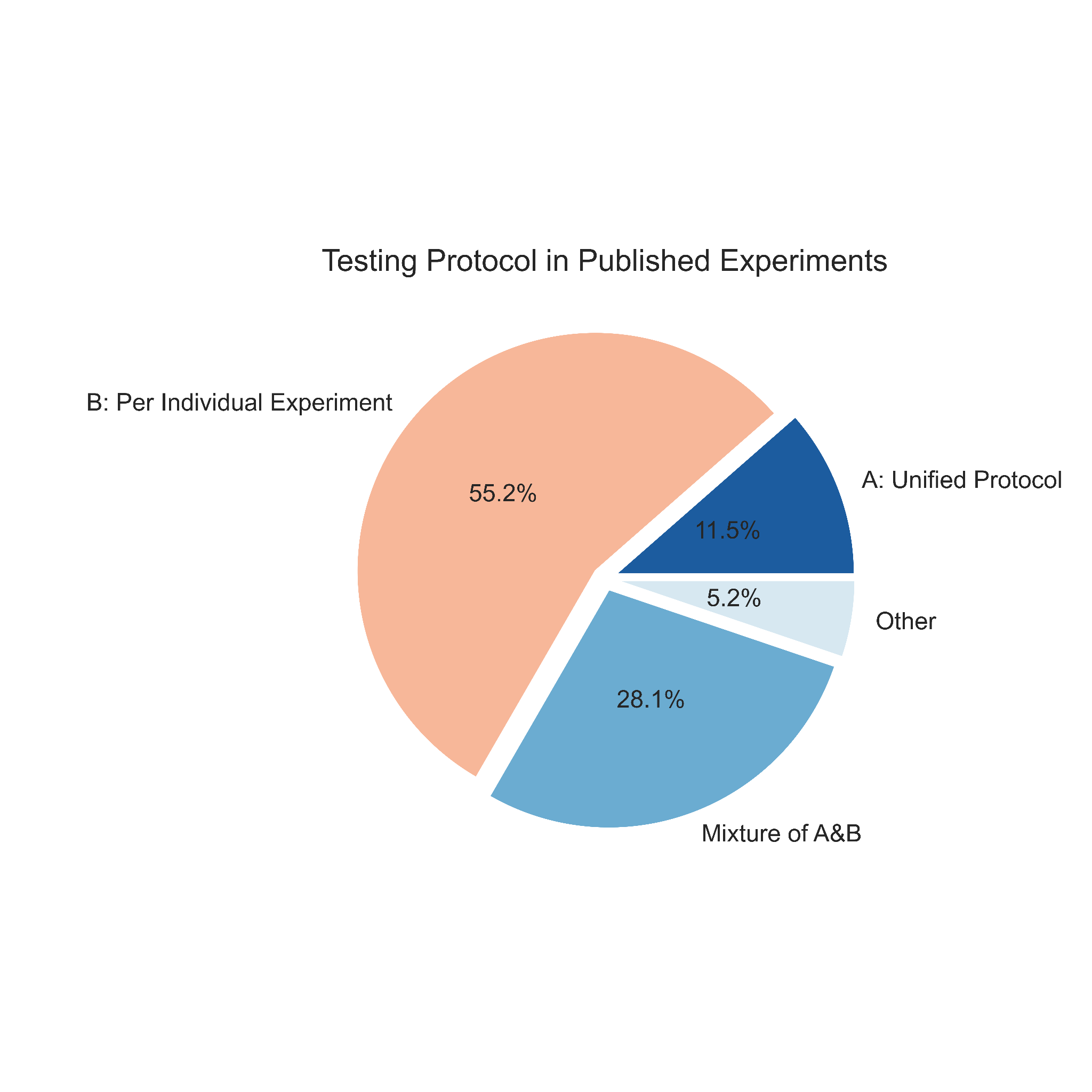


*Figure 5: Pie chart of the proportion of survey participants reporting the use of a unified protocol to test all their experiments*

1. If you did test the overall duration of your experiment, how did you go about to test it?
   1. Using a stopwatch
   2. By computing the overall duration from the log files
   3. Both
   4. I did not test the overall duration
   5. Other [free text]


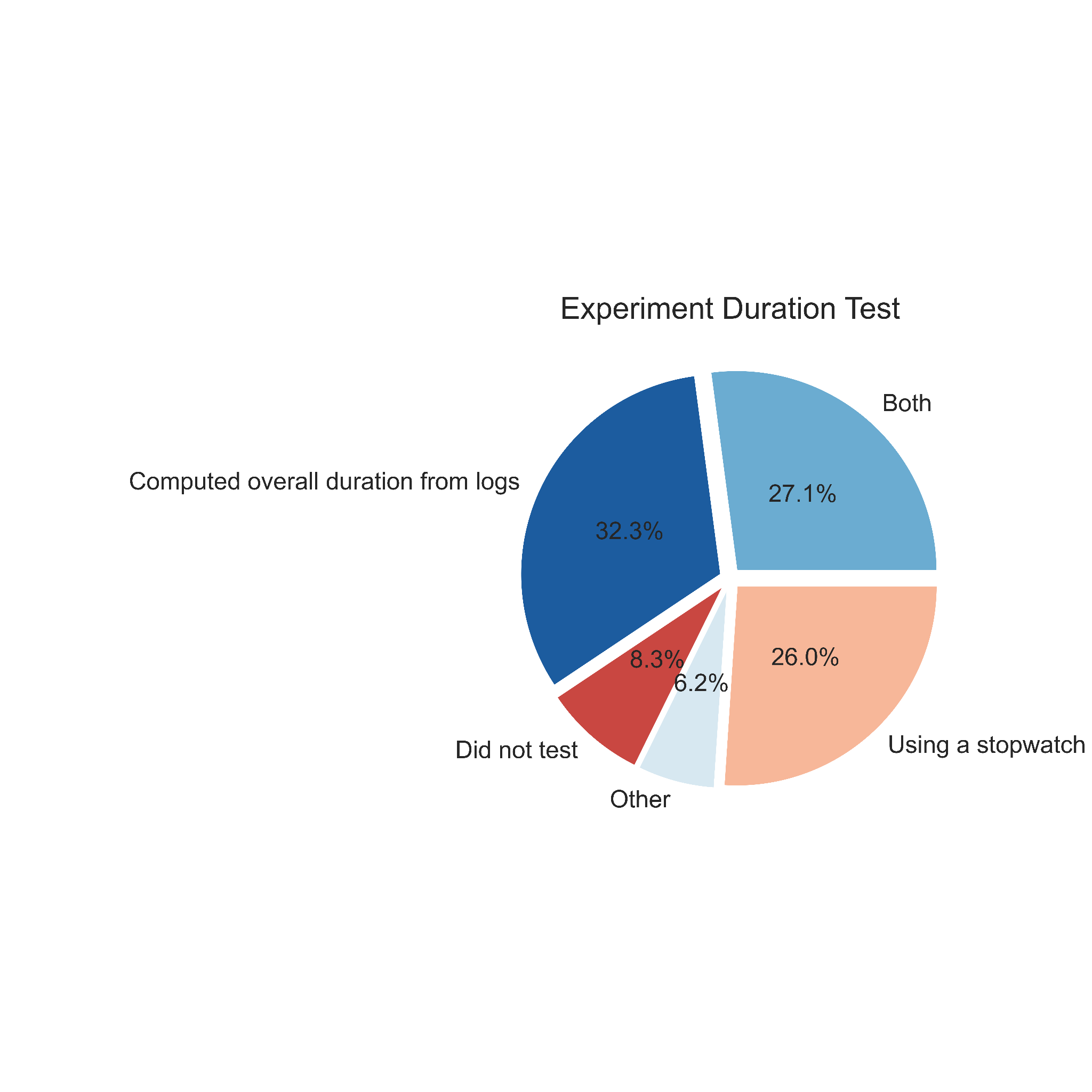


1. If you did test the on-screen vs recorded timing of events, how did you go about to test it?
   1. Using a recording device (such as a photodiode saving the signal to a file) to compute the duration of all events using a custom script
   2. By computing the duration of events from their logged timestamps (in the experiment output files)
   3. I did not test event timing
   4. Other [free text]


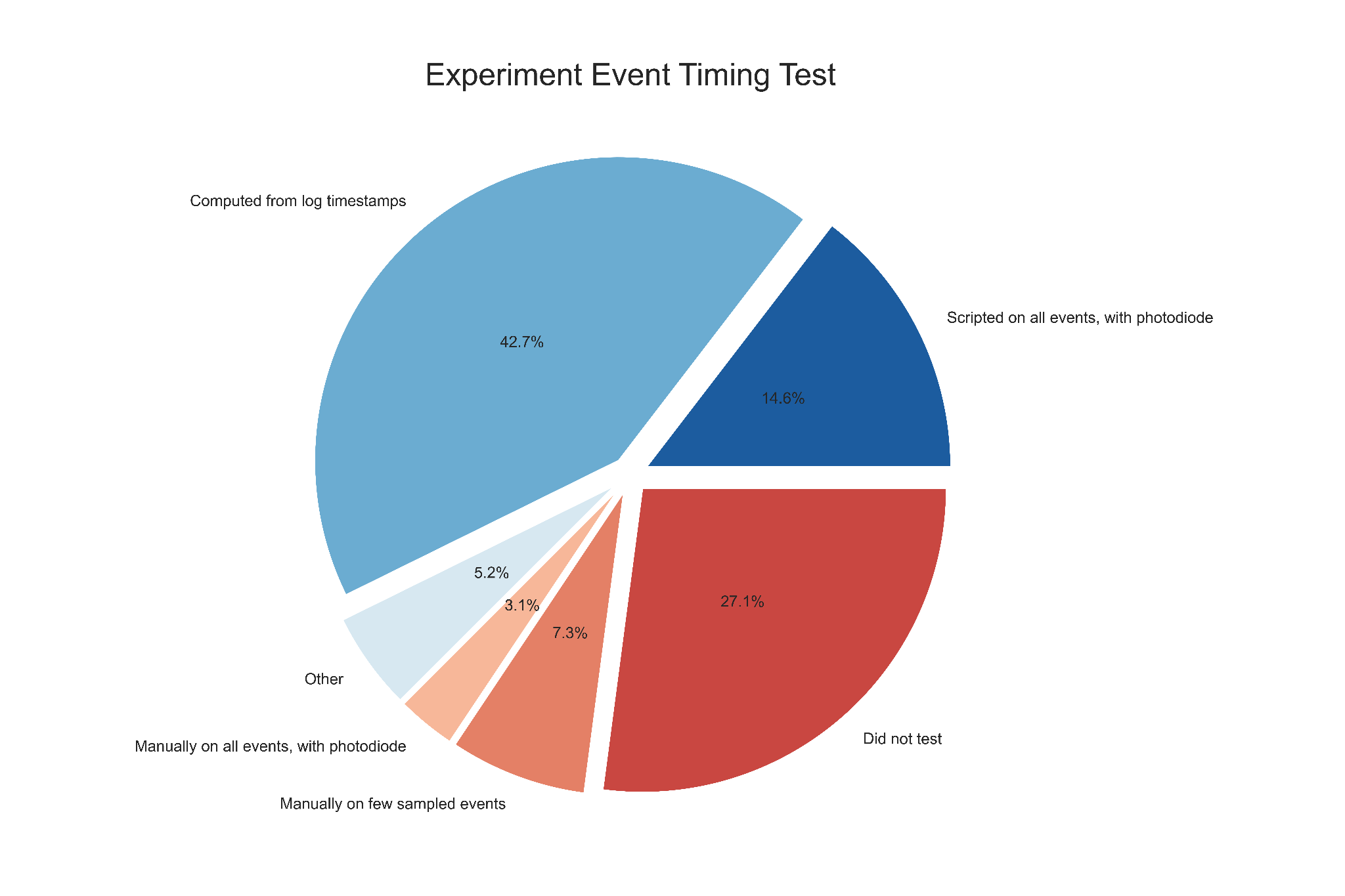


*Figure 6: Pie chart of the proportion of survery participants reporting various ways to measure the duration of events in their experiment*

1. If you did test on-screen content vs recorded content, how did you go about to test it?
   1. Manually comparing the events presented on the screen against what was recorded in the log file
   2. Comparing the recorded events with the experimental paradigm scheme
   3. I did not test the content of events


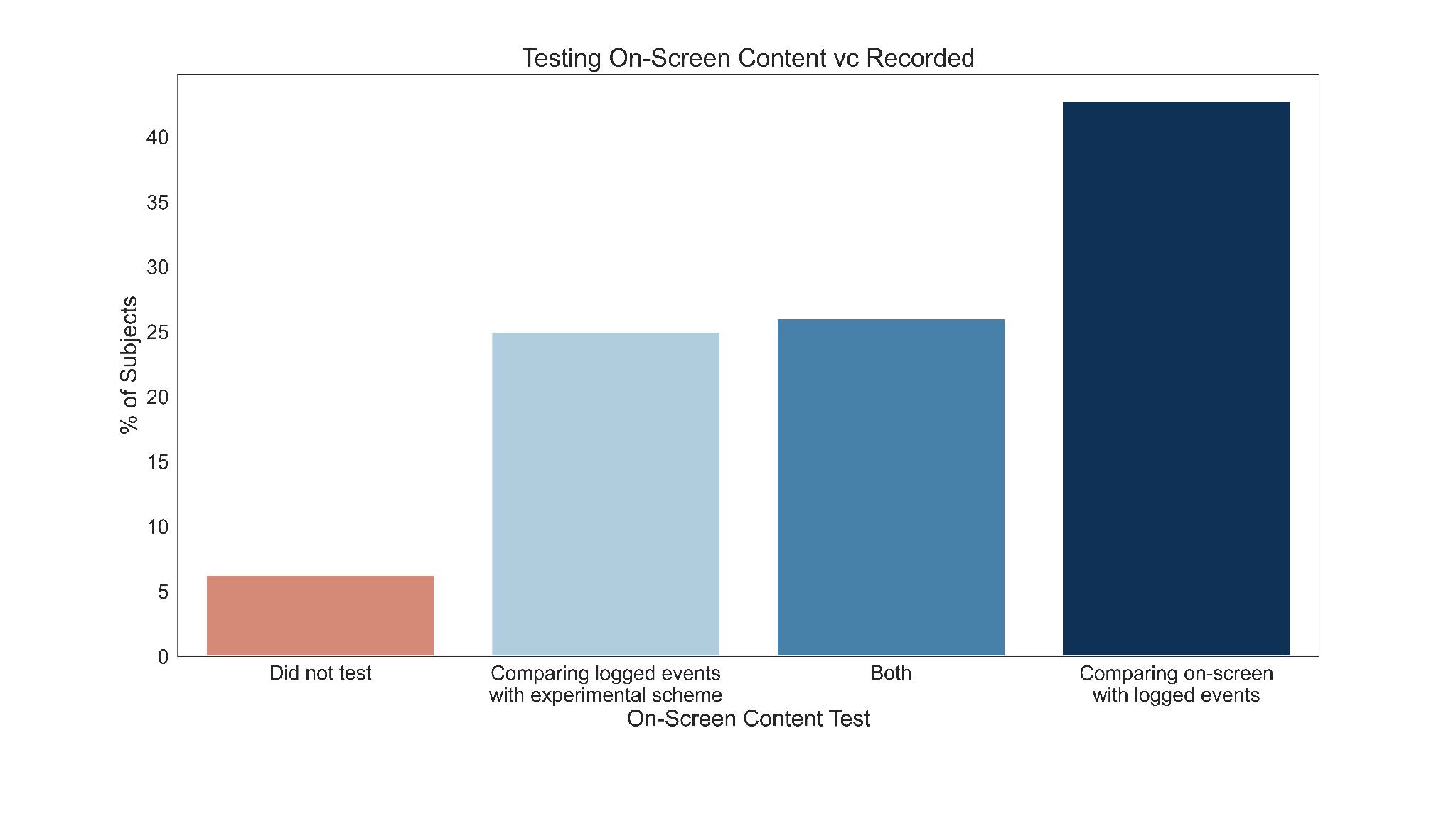


*Figure 7: Bar graph of the proportion of survey participants reporting various ways to test the validity of logged events content*

1. If you did test the pseudo-randomization scheme fulfillment, how did you go about to test it?
   1. Manually checking the log file to see if the constraints are respected
   2. Using scripts automatically investigating the log files to see if the constraints are respected
   3. I did not test the pseudo-randomization of events
   4. Other [free text]


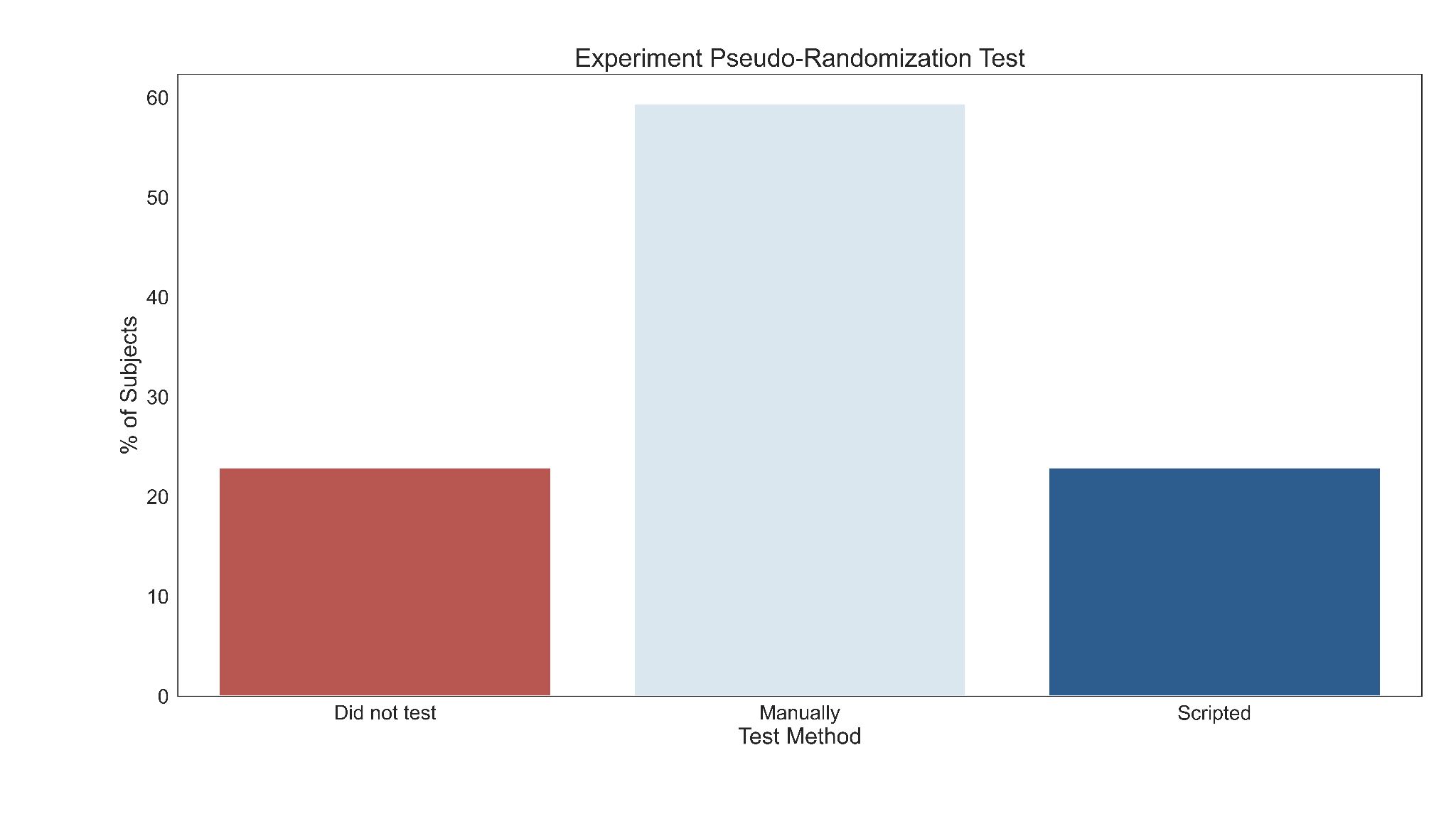


*Figure 8: Bar chart of the proportion of survey participants reporting various ways to test the randomization scheme in their experiment*

1. Did you report about the checks you performed and their results?
   1. I reported in detail the results of all the tests I performed
   2. I reported the results of some of the tests I performed
   3. I didn’t report the results of any of the tests I performed

1. If you did report the results of the checks in your publication, where were they reported? (e.g., pre-registration, supplementary material, methods...) [free text]
2. If you did not report the checks, why not?
   1. Because I did not think it is relevant
   2. Because I did not know where it should be reported
   3. Other [free text]


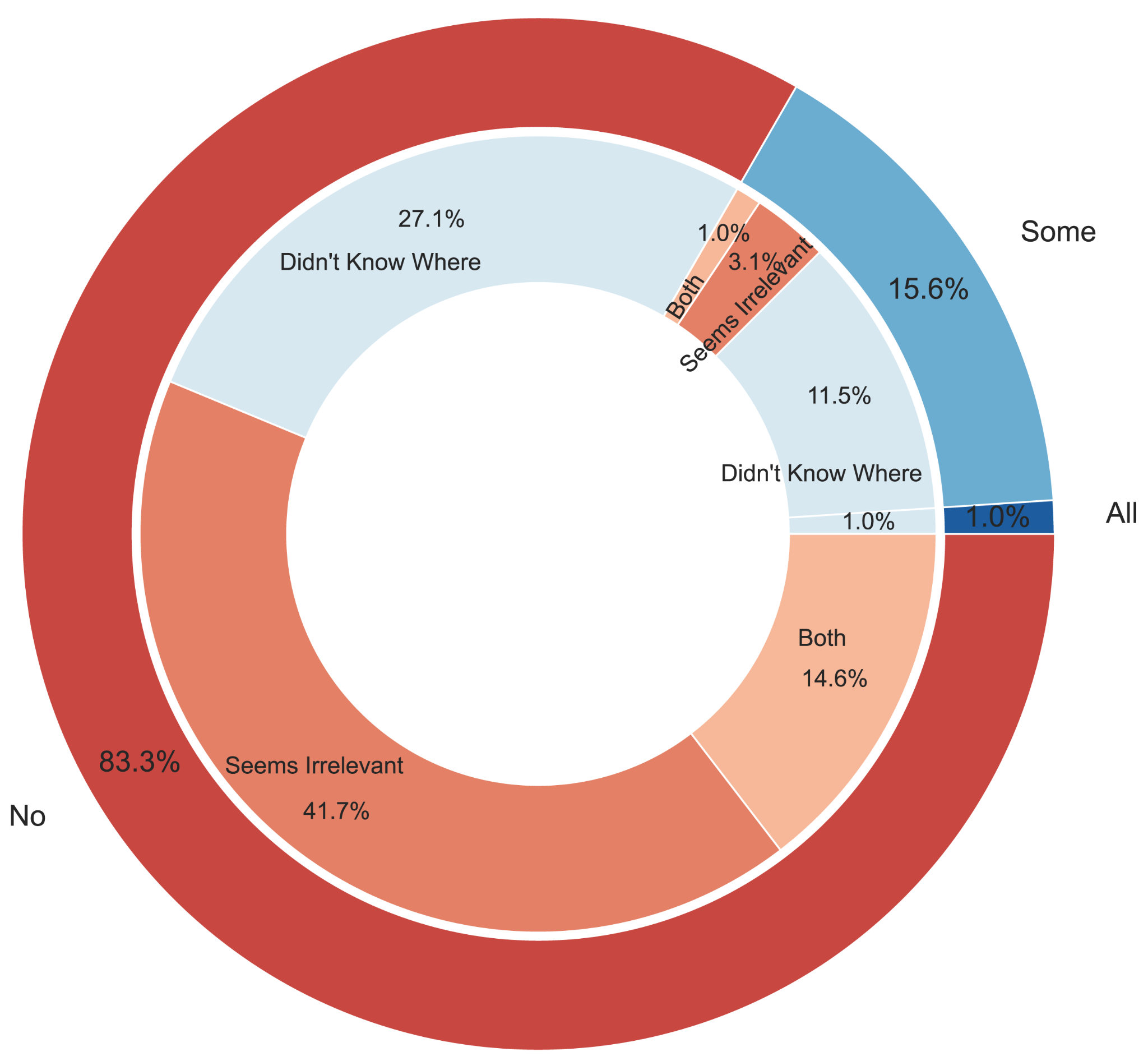


*Figure 9: Nested pie chart of the proportion of participants who reported their tests in published papers*

1. Is the test battery you performed in your last experiment replicable? (i.e., can a researcher who repeats your experiment perform all the checks without approaching you?)
   1. Yes
   2. No
   3. Other [free text]


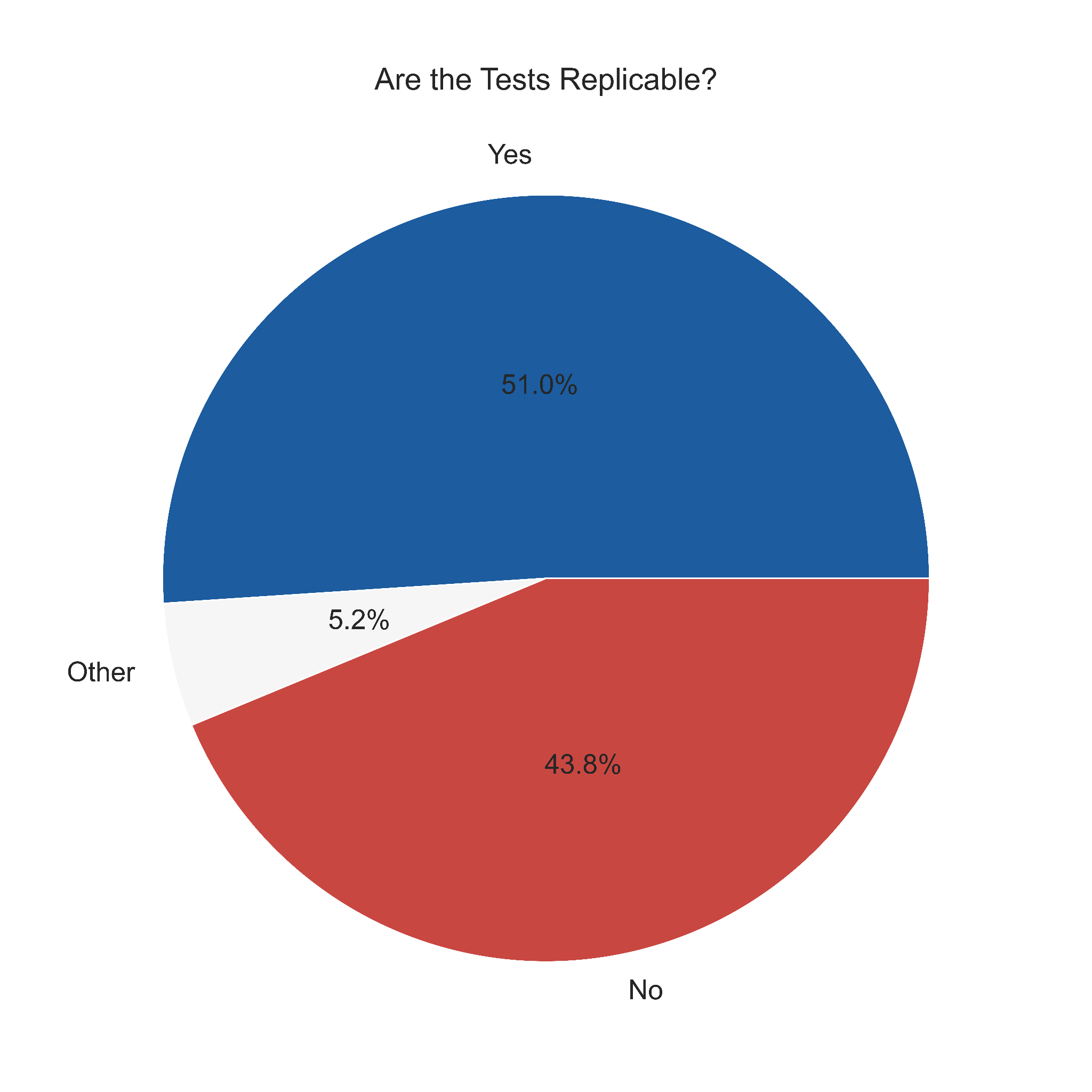


*Figure 10: Pie chart of the proportion of survey participants reporting whether the performed tests are replicable*

#### Section C: General Questions

1. In your last published experimental study, what were the subjects?
   1. Human
   2. Non-human
   3. Both
   4. Aliens


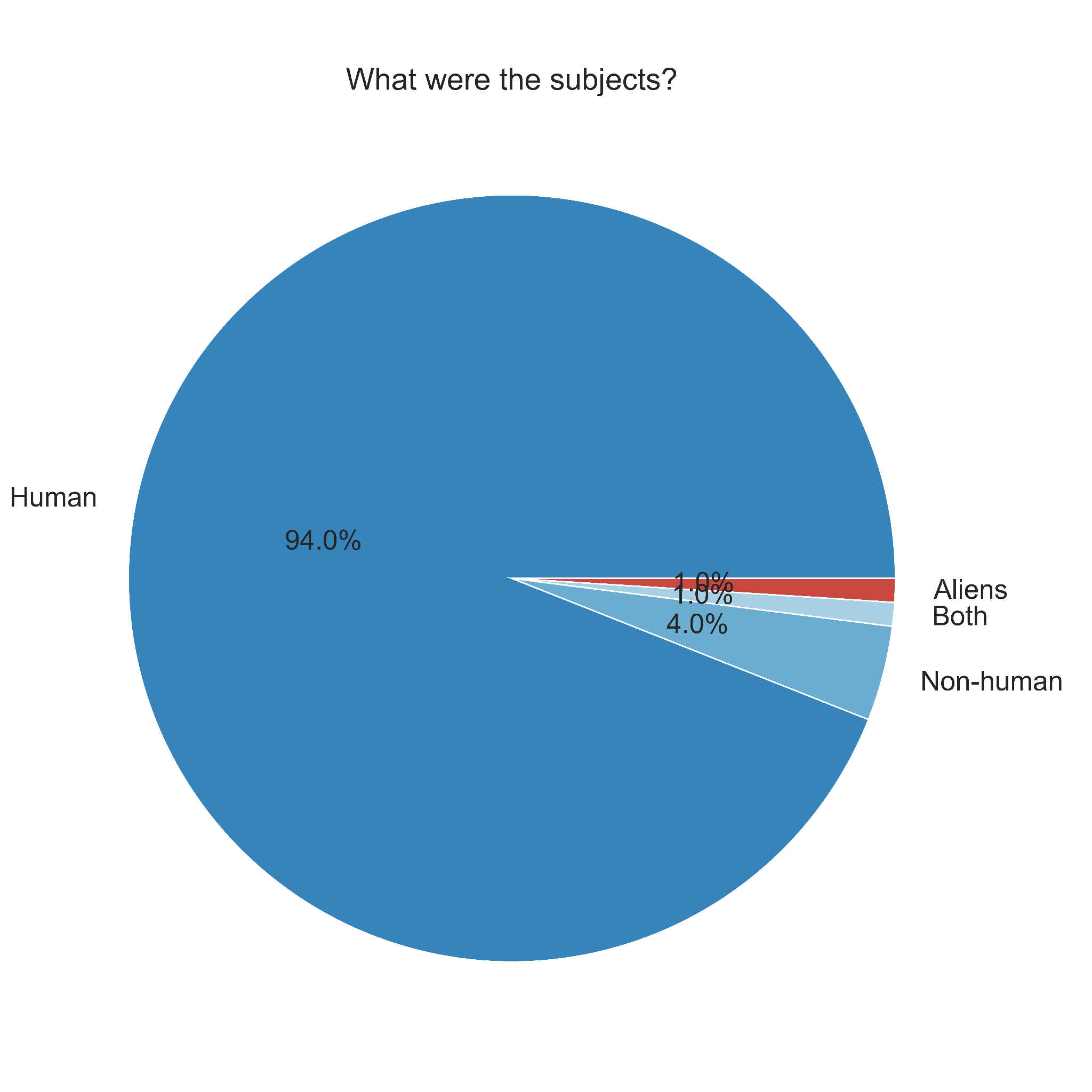


*Figure 11: Pie chart of the proportion of studies concerning particular species across surveyed participants*

Notably, ‘Aliens’ was a catch-option; however, the only participant who marked it had an otherwise valid response, which is why we chose to include them in the final sample.

1. In your last published experiment, what did you record?
   1. Behavior (e.g., report, motor responses)
   2. Gaze and eye-tracking parameters
   3. SCR/temperature/HRV
   4. EEG
   5. fMRI
   6. iEEG
   7. Other [free text]


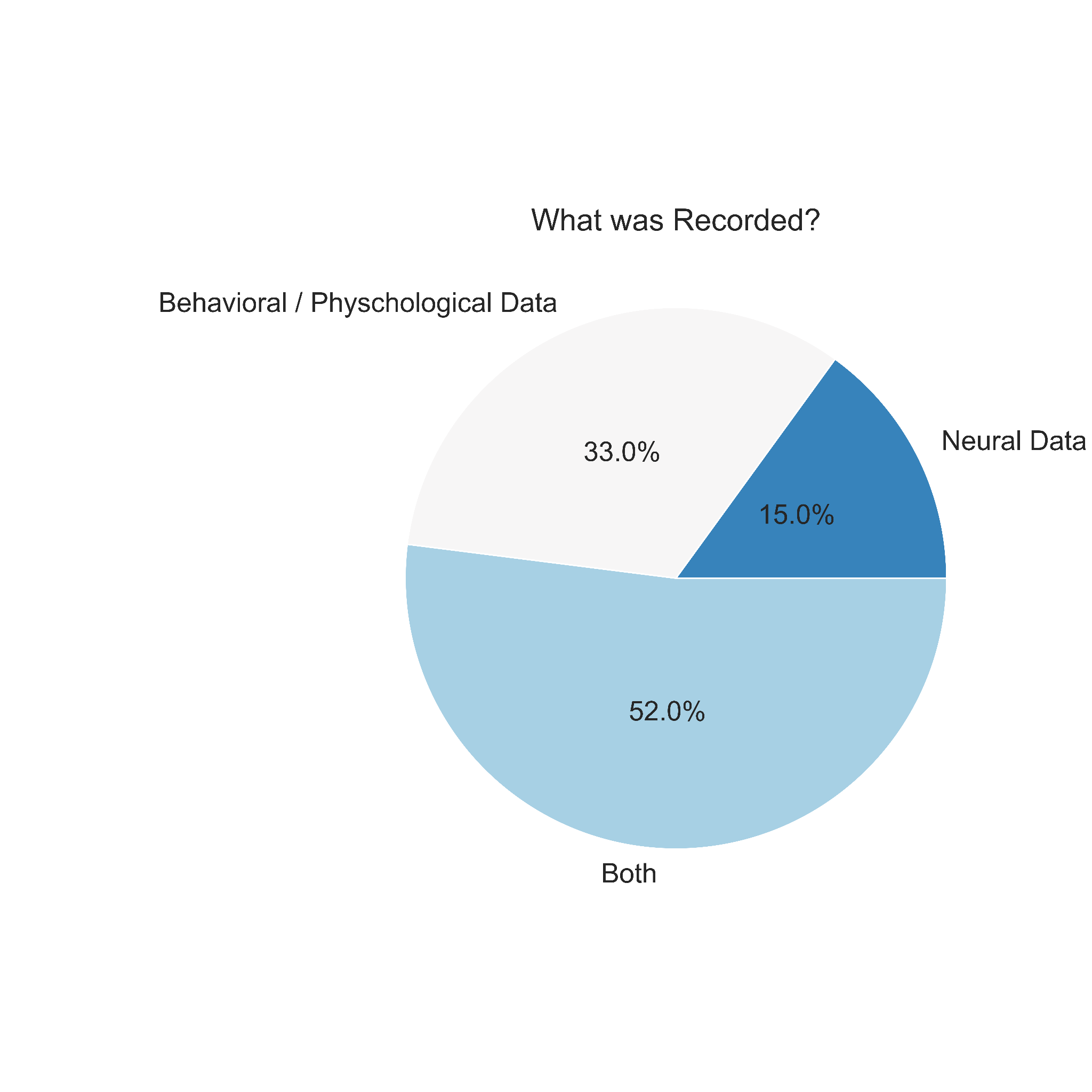


*Figure 12: Pie chart of the proportion of collected data types across the surveryed participants*

1. Have you ever noticed an issue with the data collection procedure after the data was collected, that could have been prevented by performing tests in advance? (e.g., crucial parameters were not logged or were logged incorrectly, triggers were not sent or received, responses were not recorded or recorded incorrectly)
   1. Yes
   2. No


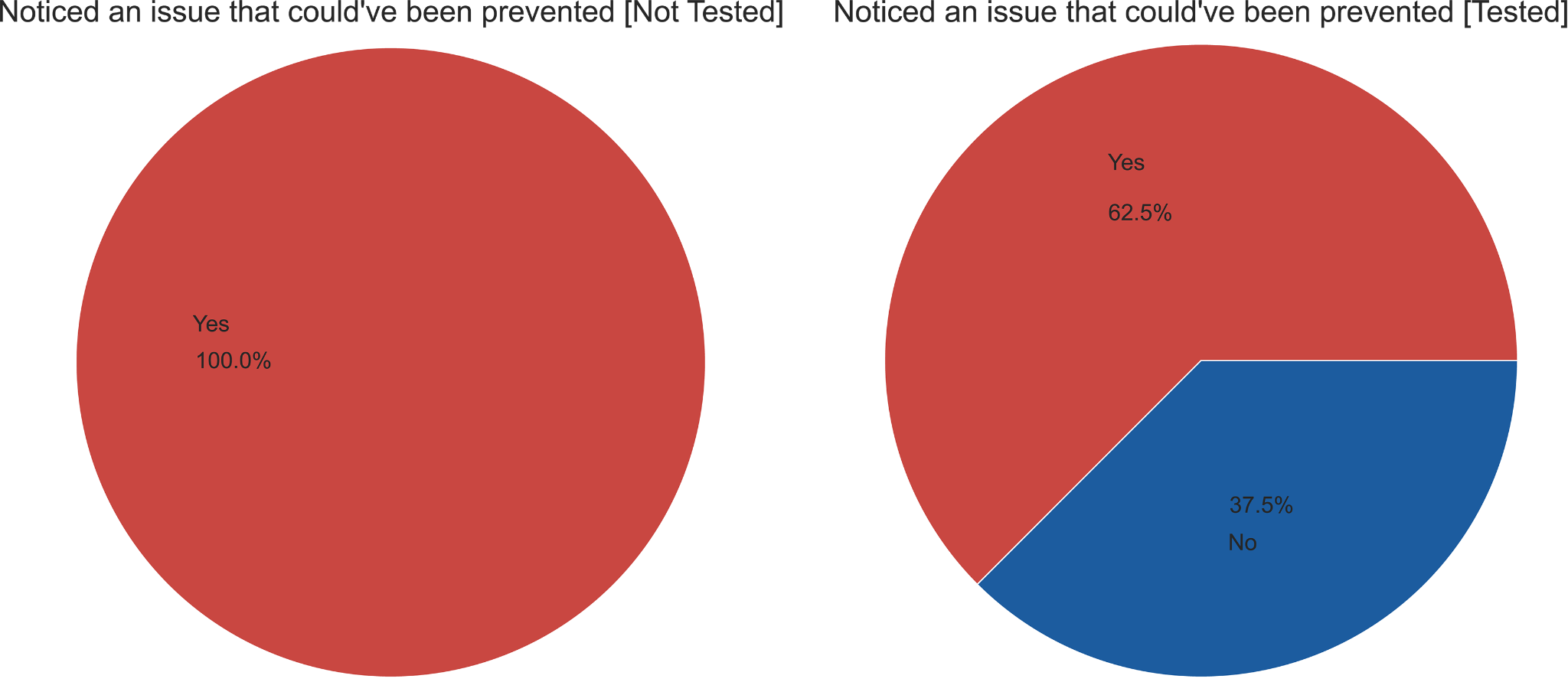


*Figure 13: Pie chart of the proportion of survey participants who reported finding issues that could have been prevented among those that did not perform any tests (left) and among those that did (right)*

1. Discipline in neuroscience:
   1. Psychology
   2. Biology
   3. Psychiatry
   4. Other [free text]


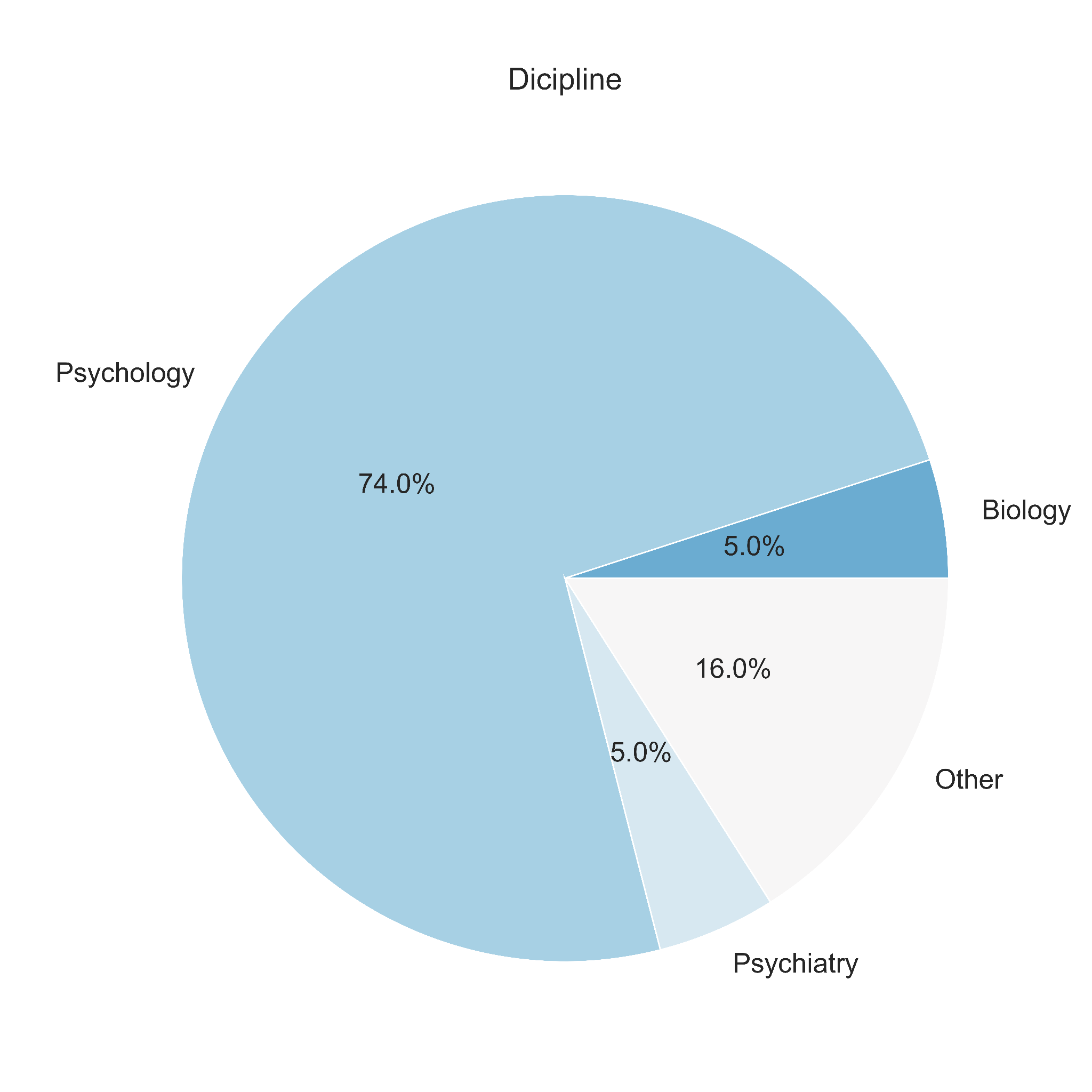


*Figure 14: Pie chart of the proportion of various disciplines represented among the surveyed participants*

1. What is your position?
   1. Undergraduate/ bachelor/ master's student
   2. Graduate student/ PhD student
   3. Post doc/senior researcher
   4. PI/Group leader
   5. Research assistant
   6. Other [free text]


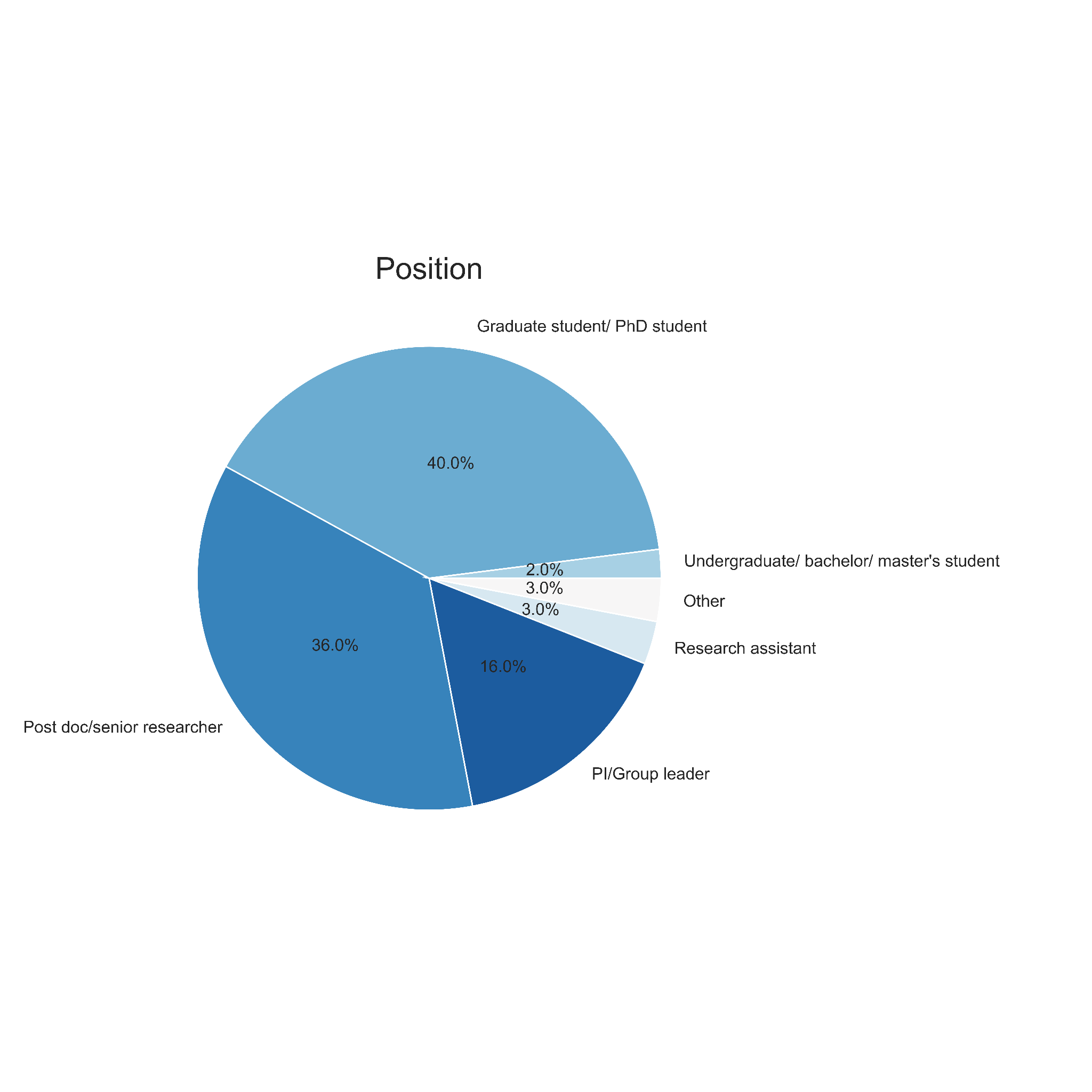


*Figure 15: Pie chart of the career stages represented in the surveyed participants*

##

## 2. Simulation

### Simulating Reaction times (RT)

A simulation was conducted to generate reaction times for two arbitrary conditions using a log normal distribution. The log normal distribution was chosen as it is commonly used to model reaction time data and has the desirable property of being positively skewed, which reflects the fact that reaction times are typically longer for slower participants. The standard deviation ($\sigma$) of the distribution was fixed to 20ms across both conditions. The mean of 450ms was used as the reference point, and the means of the two conditions were adjusted based on a desired effect size.

We simulated a difference in reaction time by inflating the mean value of condition 2 ($\mu_{cond 2}$, each condition is referred to as Condition 1 and 2 hereafter), while setting the mean of condition 1 ($\mu_{cond 1}$) to the default value of 450ms. As the reaction time is simulated using a log normal distribution, the difference between both conditions necessary to achieve the expected effect size must be observed in log space. The mean of condition 2 was therefore computed as:

**Eq. 1** $\mu_{cond 2}= e^{\theta* \sigma+ln(\mu_{cond 1})}$

Where $\theta$ refers to the simulated effect size.

In other words, the simulation was designed to generate data that reflected the desired effect size between the two conditions by adjusting the means of the log normal distributions of the second condition. We simulated 100 data points for each condition. The effect size was computed as the difference between the log transformed data of each condition divided by the pooled standard deviation:

**Eq. 2** $\hat{\theta}= \frac{ln(\mu_{cond 1})-ln(\mu_{cond 2})}{\sigma_{pooled}}$

### Simulating Event-Related Potentials (ERP)

Similarly, we simulated EEG data that reflected the P100 component, a commonly studied ERP in EEG research. The P100 component was modeled as a Gaussian function, which was fitted to an existing EEG data set (Kaneshiro et al., 2015) to determine the parameters for the Gaussian. In this data set, participants passively viewed images of different categories. The data were averaged across all trials within participants and then across participants in the channel “E77”. This electrode was selected as located posteriorly and used in several studies as part of the cluster of electrodes to compute the P100 component. The Gaussian was defined as:

**Eq. 3** $G(t) = amp * exp(-({(t-\mu)}^{2})/(2*\sigma^{2})) + C$

where G(t) represents the Gaussian function, A represents the amplitude, μ represents the mean, σ represents the standard deviation, t represents the time, and C represents the offset. The scipy.optimize.curve_fit function with the trust-region reflective (TRF) method was used to determine the parameters for the Gaussian.

To simulate a difference between two conditions of a desired effect size, the amplitude of the Gaussian of each condition was set for each trial based on a specific effect size as:

**Eq. 4** ${amp}_{cond}=amp \pm(\theta* \sigma) / 2$

while the mean, standard deviation, and offset remained the same. Here $\theta$ refers to the desired effect size and$\sigma$ reflects the noise that is added in the next step.

Using these adjusted parameters, $100$ noise free sweeps were generated for each condition, by fitting the component parameters to a linearly spaced time scale from -0.5 to 1 second at a sampling rate of 512Hz. Finally, independent and identically distributed (i.i.d.) noise was introduced at each time point in each trial, drawn from a normal distribution independent at each time point defined as:

$Noise \sim N(\mu, \sigma^{2})$

$\mu= 0$

$\sigma= k \times std({ERP}_{noise free})$

This procedure allowed for the simulation of multiple trials reflecting the desired effect size between two conditions, while also taking into account the inherent variability present in real EEG data. The *k* constant was set to 0.1, such that the signal-to-noise ratio is 10 to 1. This procedure was repeated, varying the $\theta$ parameter from 0.1 to 1 in 0.1 increments. The $\theta$parameter is based on the standardized mean difference (SMD) measure of effect size defined as:

**Eq. 5** $SMD = \frac{\mu_{1}-\mu_{2}}{\sigma}$

In our simulation, the $\sigma$ parameter is based on the overall noise in the entire time series. The difference between the two conditions is, however, introduced as a difference in peak. The variance of the peak within a given time window is lower than the variance of the overall signal. The $\theta$ parameter in our implementation, therefore isn’ the true effect size but it is proportional to it.

### Simulating malfunctioning experimental paradigms

Using the highly controlled simulated ERP, we then introduced a particular type of noise reflecting experimental paradigms artifacts. As described below, event-based paradigms are based on presenting specific contents at specific times, and then performing temporally aligned investigations of the neural responses. In such paradigms, malfunctions can either impact the timing of the event's presentation or induce a mismatch between what was presented to the participants and what was recorded as being presented to the participant or both. The former will be referred to as presentation jitter and the latter as label shuffle.

#### Simulating presentation jitter

To simulate presentation jitter, the latency of a given proportion of trials onsets was shifted by a certain duration. In a typical experimental setup, the amount of jitter is bound to be a multiple of the display’s refresh rate. Several jitter durations were introduced to investigate such differences in experimental hardwares. The selected jitter durations were varied from 4 to 40 ms in 4ms increments, to represent most of the commonly available displays refresh rates. In addition, depending on how severe the experimental malfunction is, the proportion of trials presenting the jitter will differ. In order to model different degrees of impairment, the proportion of affected trials was varied from 5 to 40% in 5% increments.

#### Simulating label shuffle

Experimental code might fail with regards to the timing of the events but also with regards to the labeling of the trials (e.g., when presenting an experimental condition or stimulus that is different from the originally intended one). To evaluate the impact of mislabeled trials on the final results, trial labels were shuffled between conditions, and the proportion varied from 5% to 40% in 5% increments. This process was repeated 100 times for each increment, to ensure robustness of the results to the random selection of trials to which the effect must be introduced.

#### Analyzing the impact of malfunctions on experimental setups

The simulated data were analyzed in the P100 time window between 80 and 140 ms following stimulus onset. In this time window, peak amplitude (maximum value within time window), peak latency (maximum value latency) and average amplitude over the entire time window (80-140 ms) were computed. A t-statistic on the difference between conditions was computed based on the average and peak measures separately.

# Bibliography

Kaneshiro, B., Guimaraes, M. P., Kim, H.-S., Norcia, A. M., & Suppes, P. (2015). A Representational Similarity Analysis of the Dynamics of Object Processing Using Single-Trial EEG Classification. *PLOS ONE*, *10*(8), e0135697. <https://doi.org/10.1371/journal.pone.0135697>
